# Effects of transcranial direct current stimulation on patient-reported outcomes in chronic low back pain: a systematic review and three-level meta-analysis

**DOI:** 10.3389/fmed.2026.1950897

**Published:** 2026-10-07

**Authors:** Chenming Wang, Nianting Zhou, Sizhuo Zhang, Peng Chen

**Affiliations:** 1 Wuhan Business University, Wuhan, Hubei, China; 2 Department of Rehabilitation Medicine, Shanghai Public Health Clinical Center, Fudan University, Shanghai, China; 3 School of Exercise and Health, Shanghai University of Sport, Shanghai, China

**Keywords:** chronic low back pain, disability, meta-analysis, pain, psychosocial outcomes, transcranial direct current stimulation

## Abstract

**Background:**

Growing evidence suggests that central nervous system alterations may contribute to the persistence of chronic low back pain (CLBP). Transcranial direct current stimulation (tDCS) can effectively modulate cortical excitability and holds considerable potential for the rehabilitation of CLBP. However, evidence regarding its effects remains controversial.

**Purpose:**

This study aimed to systematically evaluate the effects of tDCS on pain, disability, and psychosocial outcomes in individuals with CLBP.

**Methods:**

Five electronic databases were searched from their inception to January 20, 2026. Randomized controlled trials were included to evaluate the effects of tDCS in CLBP individuals. Meta-analysis was performed using the random-effects model implemented in the metafor package in R. Subgroup analyses were based on tDCS polarity, electrode placement, current intensity, treatment sessions, and comparison type.

**Results:**

A total of 14 randomized controlled trials were included. Meta-analysis results showed that compared with the control group, the tDCS group reported significantly reduced pain (standardized mean difference [SMD] = −0.40, 95% confidence interval [CI]: −0.61 to −0.19, *P* < 0.001) and disability (SMD = −0.18, 95 CI: −0.34 to −0.03, *P* < 0.02) in individuals with CLBP. However, no significant effects were observed for follow-up pain (SMD = −0.19, 95% CI: −0.46–0.08, *P* = 0.173), follow-up disability (SMD = −0.10, 95% CI: −0.26–0.05, *P* = 0.194), quality of life (SMD = 0.17, 95% CI: −0.08–0.41, *P* = 0.179), anxiety (SMD = −0.04, 95% CI: −0.23–0.15, *P* = 0.659), or depression (SMD = −0.18, 95% CI: −0.49–0.12, *P* = 0.241). Subgroup analyses based on tDCS polarity, electrode placement, current intensity, treatment sessions, and comparison type showed variations in effect estimates, but no significant between-subgroup differences were detected.

**Conclusion:**

Current evidence suggests that tDCS may reduce pain and produce a small improvement in disability in individuals with CLBP. Given the low-to-moderate certainty of the available evidence, these findings should be interpreted cautiously.

**Systematic Review Registration:**

identifier CRD420261286288.

## 1 Introduction

Low back pain (LBP) is a major global health challenge (1, 2) and is currently the leading cause of years lived with disability worldwide (3, 4). The global prevalence of LBP exceeded 600 million in 2020, and over 800 million individuals worldwide are projected to suffer from LBP by 2050 (1). A substantial proportion of individuals with LBP experience persistent symptoms, and pain lasting for at least 12 weeks is generally classified as chronic LBP (CLBP), which can be associated with recurrent pain, disability, and substantial socioeconomic burden (2, 5). Additionally, individuals with CLBP often exhibit psychosocial impairments (such as anxiety and depression) (6), which further exacerbates their pain symptoms (7, 8). Current clinical guidelines recommend a stepped-care approach for the treatment of CLBP, starting with nonpharmacological approaches, followed by nonopioid medications, and then opioid medications only when the potential benefits clearly outweigh the risks (9, 10). This treatment hierarchy further highlights the importance of developing safe, effective, and mechanism-based nonpharmacological treatments for CLBP.

Accumulating evidence suggests that CLBP is associated with structural and functional alterations in multiple brain regions involved in pain perception, modulation, and affective processing (11, 12). Such maladaptive neuroplastic changes may contribute to the persistence and maintenance of pain (13, 14), highlighting the central nervous system as a potentially important therapeutic target in CLBP (15). Interventions that directly modulate brain regions involved in pain processing may be effective in CLBP treatment (16). Transcranial direct current stimulation (tDCS) is a widely utilized, safe, and reliable neuromodulation technique (17) that can effectively regulate cortical excitability by altering the resting membrane potential of intracortical neurons and reducing depolarization thresholds, ultimately improving brain function (18, 19). However, whether these neurophysiological effects translate into consistent clinical benefits for individuals with CLBP remains uncertain.

In 2020, a meta-analysis quantitatively analyzing five randomized controlled trials (RCTs) indicated that tDCS had no significant effect on pain and disability in individuals with CLBP (20). However, this meta-analysis was confined to pain and disability outcomes and included a limited number of studies, and thus its findings are insufficiently robust. Additionally, this study did not examine the impact of tDCS parameters (such as polarity, electrode placement, current intensity, and treatment sessions) on outcomes or potential moderating factors. The therapeutic efficacy of tDCS for CLBP and the optimal treatment parameters remain unclear, indicating a considerable gap in current evidence.

The objective of this review is to conduct a systematic review and subsequent meta-analysis to comprehensively analyze the effects of tDCS on pain, disability, quality of life anxiety, and depression, in individuals with CLBP and to explore potential moderating factors and potentially effective stimulation parameters. Our findings may help inform the optimization of rehabilitation strategies and provide updated evidence for future research and clinical practice.

## 2 Methods

### 2.1 Protocol and registration

This review complied with the Preferred Reporting Items for Systematic Reviews and Meta-Analyses (PRISMA) statement (21) outlined in the guidelines. The study was registered with the International Prospective Register of Systematic Reviews (Registration No. CRD420261286288). Supplementary Table S1 lists the PRISMA checklist. As this study solely involved the review and synthesis of existing literature, approval from an institutional review board was not required.

### 2.2 Search strategy

A systematic search was conducted across PubMed, Cochrane Library, Embase, Web of Science, and EBSCO databases to identify research articles evaluating the effects of tDCS on individuals with CLBP. All relevant articles published globally in any language were included from database inception to January 20, 2026, and no restriction on publication date was implemented. The search was conducted using a combination of subject headings and keywords. Search terms included “back pain,” “low back pain,” “chronic low back pain,” “CLBP,” “transcranial direct current stimulation,” and “tDCS.” The detailed search strategy is presented in Supplementary Table S1.

### 2.3 Inclusion and exclusion criteria

The inclusion criteria were as follows: (1) population: individuals aged ≥18 years with LBP persisting for ≥3 months; LBP was defined as pain, muscle tension, or stiffness located below the costal margin and above the inferior gluteal folds, with or without leg pain (sciatica); (2) intervention: tDCS was applied either alone or in combination with conventional rehabilitation; (3) comparison: sham tDCS or conventional rehabilitation or both were employed; (4) outcome measures: primary outcome measures included pain, follow-up pain, disability, and follow-up disability; secondary outcome measures encompassed quality of life, anxiety, and depression; (5) study type: RCTs. The exclusion criteria were as follows: (1) studies targeting individuals with acute, subacute LBP or those who had undergone lumbar spine surgery; (2) studies failing to report relevant outcome measures; (3) case reports or descriptive studies, or studies not published as peer-reviewed journal articles, including theses, book chapters, and conference abstracts; (4) for duplicate publications, that is, when two studies by the same author reported identical data for the same variables and groups across different publications, duplicates were removed and a single record was used for analysis.

### 2.4 Study selection and data extraction

Literature screening was conducted independently by two authors (NTZ and SZZ). Potential discrepancies regarding the inclusion or exclusion criteria were resolved through discussion with a third author (PC). The articles were screened and managed using EndNote X9 software, and all duplicate articles were removed. Reference lists of included studies and relevant reviews were screened by one author to identify additional studies relevant to this review (CMW). Data extraction was performed independently by two researchers (NTZ and SZZ), and a third researcher was consulted in cases of disagreement (PC). The following data were extracted from the included articles: (1) author names and publication year; (2) region of publication; (3) participants’ age and gender; (4) intervention methods for the experimental and control groups; (5) tDCS intervention parameters, including polarity, electrode placement, current intensity, and treatment sessions; (6) outcome measures, including pain, disability, and psychosocial outcomes pre- and post-intervention. For pain and functional outcomes, we extracted follow-up data; (7) adverse effects/side effects.

### 2.5 Quality assessment

The two independent reviewers used the Cochrane Risk of Bias Tool version 2 to assess the risk of bias in the included studies (22). Then, they summarized the overall quality of evidence with the summary of findings table generated using the Grading of Recommendations Assessment, Development, and Evaluation (GRADE) framework (23). Certainty of evidence refers to the confidence that the true effect lies within a particular range (24). Evidence quality was downgraded when significant deficiencies existed in risk of bias, inconsistency, imprecision, or publication bias. All study assessments were conducted independently by two authors. Any discrepancy was resolved through discussion with a third independent author.

### 2.6 Statistical analysis

All data were statistically analyzed and presented using R software (version 4.3.2) (25). Because some included studies reported multiple intervention arms or multiple outcome measures, conventional two-level meta-analytic models may violate the assumption of independence among effect sizes, potentially leading to overestimated precision due to the hierarchical and dependent structure of the data (26). To address this issue, we employed a three-level random-effects meta-analytic framework (26). This model partitions the total variance into three components: Level 1 represents sampling variance, Level 2 represents within-study variance, and Level 3 represents between-study variance, thereby accounting for dependency among effect sizes and the hierarchical structure of the data (27). To obtain robust statistical inference in the presence of residual within-study dependence, cluster-robust variance estimation with small-sample corrections was additionally applied (28). Rather than averaging or excluding multiple effect sizes from the same study, all eligible effect sizes were retained, which preserves available information and may improve statistical efficiency and precision. Model parameters were estimated using restricted maximum likelihood, and maximum likelihood estimation was additionally used as a sensitivity check to assess the robustness of the findings (29).

Given that outcomes were continuous variables, summary effect estimates were calculated using the change in values from baseline to post-intervention, their standard deviations, and the total number of participants. When standard deviations were not provided, they were calculated using standard errors or 95% confidence intervals (CI) (30). When such data were unavailable, we derived means and standard deviations from sample sizes, medians, interquartile ranges, and minimum and maximum values (31). When data were reported only in graphical format, numerical values were extracted using GetData Graph Digitizer, a validated tool for extracting data from figures (32). Additionally, a correlation coefficient of 0.5 was assumed in the calculation of the standard deviation of changes from pre- to post-intervention. This assumption was based on the moderate degree of measurement reproducibility commonly accepted in the literature and was intended to balance potential variability between pre- and post-measurements while ensuring conservative and reliable results (30). To rigorously ensure our findings were not driven by this assumption, we conducted sensitivity analyses utilizing alternative plausible correlations (*r* = 0.3 and *r* = 0.7). Pooled effects were expressed as standardized mean differences (SMD) with 95% CI. Statistical significance was considered when the 95% CI of the SMD did not cross zero. The effects were considered negligible for SMD < 0.2, small for 0.2 ≤SMD < 0.5, moderate for 0.5 ≤SMD < 0.8, and high for SMD ≥ 0.8.

The percentage of variability in the pooled estimate attributable to heterogeneity beyond chance was evaluated using *I*^2^ statistic (33), which was interpreted as follows: 0%–25%, low heterogeneity; >25%–50%, moderate heterogeneity; >50%–75%, substantial heterogeneity; and >75%–100%, considerable heterogeneity. Statistical heterogeneity among trials was examined using the Cochran Q statistic, which tests the null hypothesis of no difference in effect sizes across studies (30). The potential for publication bias was assessed using funnel plots (34) and Egger's regression test (35, 36) for outcomes including at least 10 independent studies. Sensitivity analyses were performed for each outcome by sequentially removing individual studies. Subgroup analyses based on tDCS polarity, electrode placement, current intensity, treatment sessions, and comparison type were prespecified and considered exploratory. Between-subgroup differences were assessed using omnibus tests of moderators. For pain and disability follow-up outcomes, we performed subgroup analyses stratified by follow-up time points. Meta-regression analyses were conducted within a three-level random-effects framework using restricted maximum likelihood estimation to explore potential sources of heterogeneity, with multiple effect sizes nested within studies to account for within-study dependency. Meta-regression was performed only when at least 10 independent studies were available for the corresponding model (37).

## 3 Results

### 3.1 Study selection

Electronic database and manual searches yielded 1,385 articles. After 280 duplicates were removed and 933 studies were excluded through title and abstract screening, 57 full-text articles were assessed for eligibility. Finally, 14 RCTs that fully met the inclusion criteria and were included in qualitative synthesis and meta-analysis (Figure 1).

**Figure 1:**
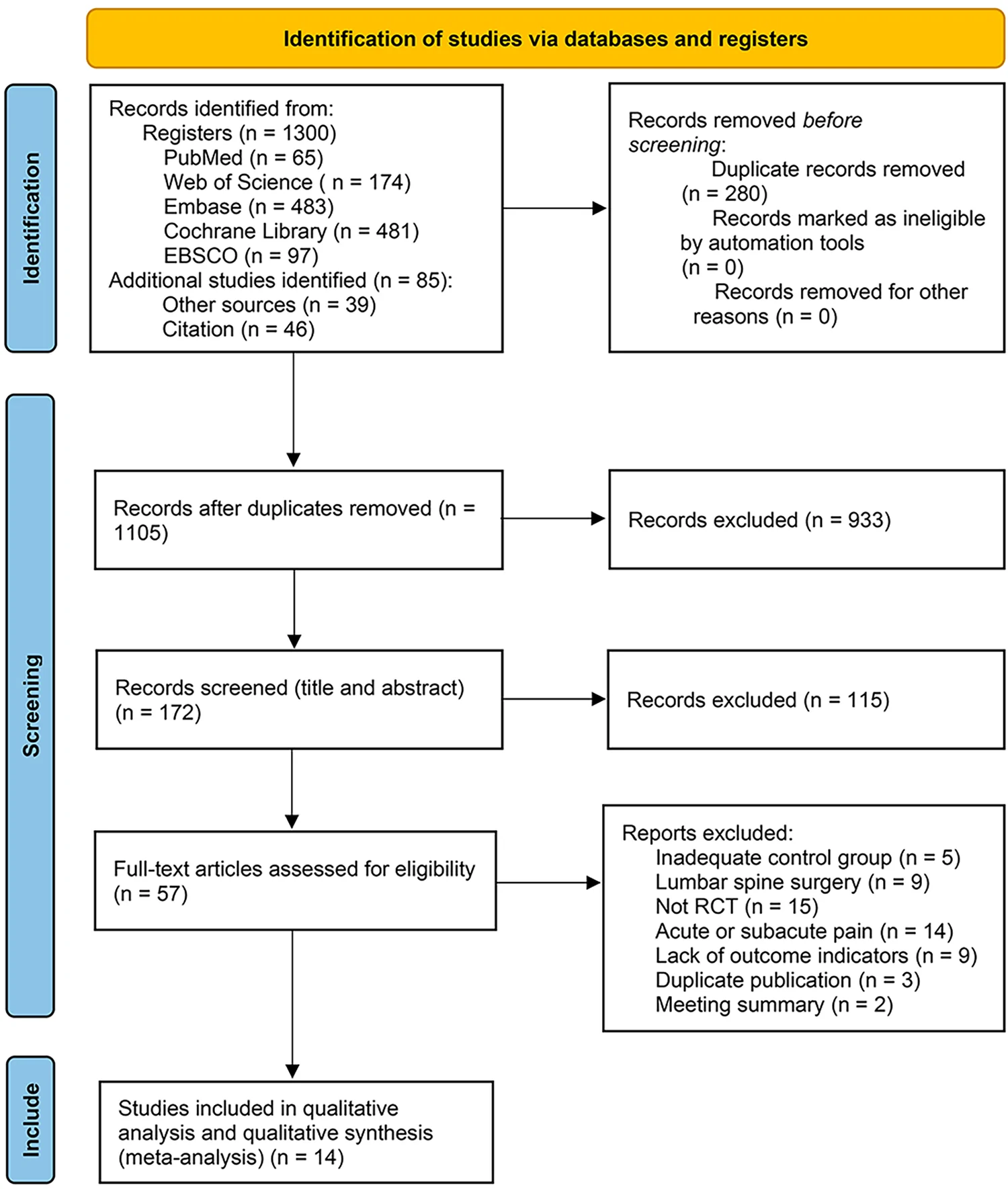
PRISMA flow diagram.

### 3.2 Characteristics of included literature

Our review included a total of 613 participants (318 in the experimental group and 295 in the control group). The mean age of participants was 44.0 years. Regarding tDCS intervention parameters, 12 studies applied anodal tDCS (38–49), whereas two studies used cathodal tDCS (50, 51). Primary motor cortex (M1) was the most frequently targeted site, which was reported in 10 studies (39, 41–49), followed by dorsolateral prefrontal cortex (DLPFC, 3 studies) (38, 40, 41), dorsal anterior cingulate cortex (dACC, 1 study) (50), and dual-site protocol targeting M1 and primary somatosensory cortex (S1) (51). Current intensity ranged from 1.5 mA to 2 mA. The number of treatment sessions ranged from 1 to 12. Detailed descriptions of participant and intervention characteristics are provided in Supplementary Table S2.

Regarding outcome measures, pain intensity was assessed using the numerical pain rating scale (38, 41–43), visual analog scale (39, 40, 44–46, 49, 51), brief pain inventory (41, 47), and defense and veterans pain rating scale (50). Disability was evaluated using the Roland–Morris disability questionnaire (39–42, 47, 49, 50), Oswestry Disability Index (ODI) (46, 51), and modified ODI (38, 48). Quality of life was measured using the 36-item short form health survey (41, 48), EuroQol-5 dimension (39, 49), RAND 36 (46), and World Health Organization Quality of Life Scale (45). Anxiety was assessed using the Depression Anxiety Stress Scale (DASS) (47), Hospital Anxiety and Depression Scale (HADS) (46), Generalized Anxiety Disorder-7 (50), State-Trait Anxiety Inventory (41), and Pain Anxiety Symptoms Scale (50). Depression levels were assessed using the Beck's Depression Inventory (41), DASS, HADS (46), and 9-item Patient Health Questionnaire (49).

### 3.3 Quality assessment

Among the 14 included studies, 8 studies (38, 39, 42, 43, 46, 49–51) (57.1%) were classified as low risk, and 6 studies (40, 41, 44, 45, 47, 48) (42.9%) were judged as having some concerns (Supplementary Figure S1). All the studies detailed their randomization procedures, primarily utilizing computer-generated random sequences or random number tables. Nine studies (38, 39, 41–43, 46, 49–51) reported no deviations from intended interventions. No missing outcome data were reported in 14 studies, reflecting a low risk in this aspect. All studies were judged to be at low risk of bias in the measurement of the outcome domain, and ten studies (38–40, 42–44, 46, 49–51) were judged to be at low risk of bias in the selection of the reported result (Supplementary Figure S2). According to the GRADE system, low-to-moderate certainty was demonstrated across all outcomes in the direct comparisons. The certainty of the evidence is summarized in Supplementary Table S3. The primary reasons for downgrading were inconsistency and imprecision.

### 3.4 Meta-analysis results

#### 3.4.1 Pain

Figure 2 and Supplementary Figure S3 present the results of the meta-analysis for pain. The outcome measure included 25 pairwise comparisons, and moderate heterogeneity was found across studies (*I*^2^ = 45.2%). Meta-analysis results demonstrated that tDCS significantly reduced pain in individuals with CLBP compared with the control group (SMD = −0.40, 95% CI: −0.61 to −0.19, *P* < 0.001). Subgroup analyses showed variations in effect estimates according to polarity, electrode placement, current intensity, number of treatment sessions, and comparison type; however, no significant between-subgroup differences were detected (Figure 3).

**Figure 2:**
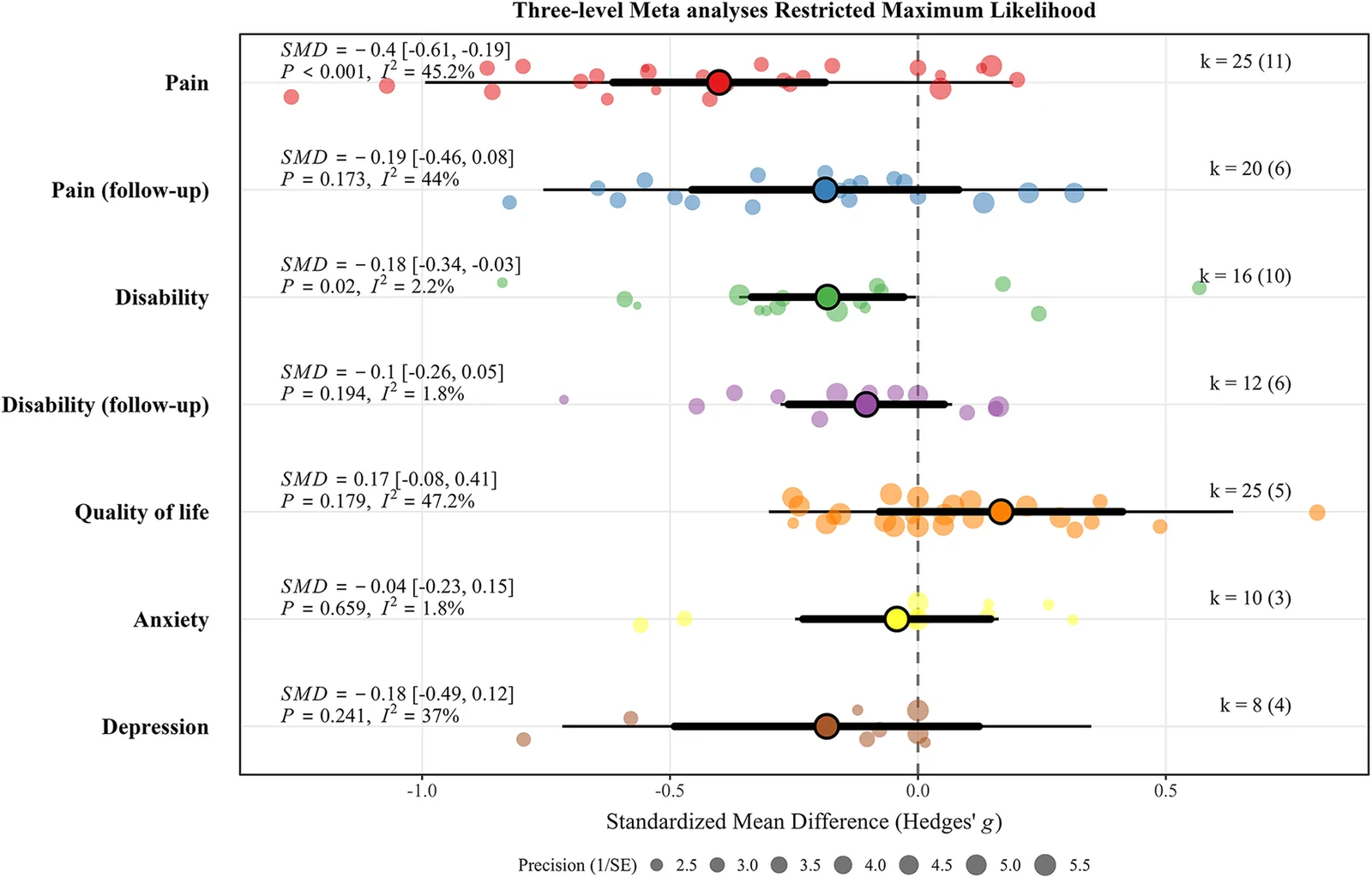
Summary of pooled effect sizes. K, the total number of effects included in the pooled effect size.

**Figure 3:**
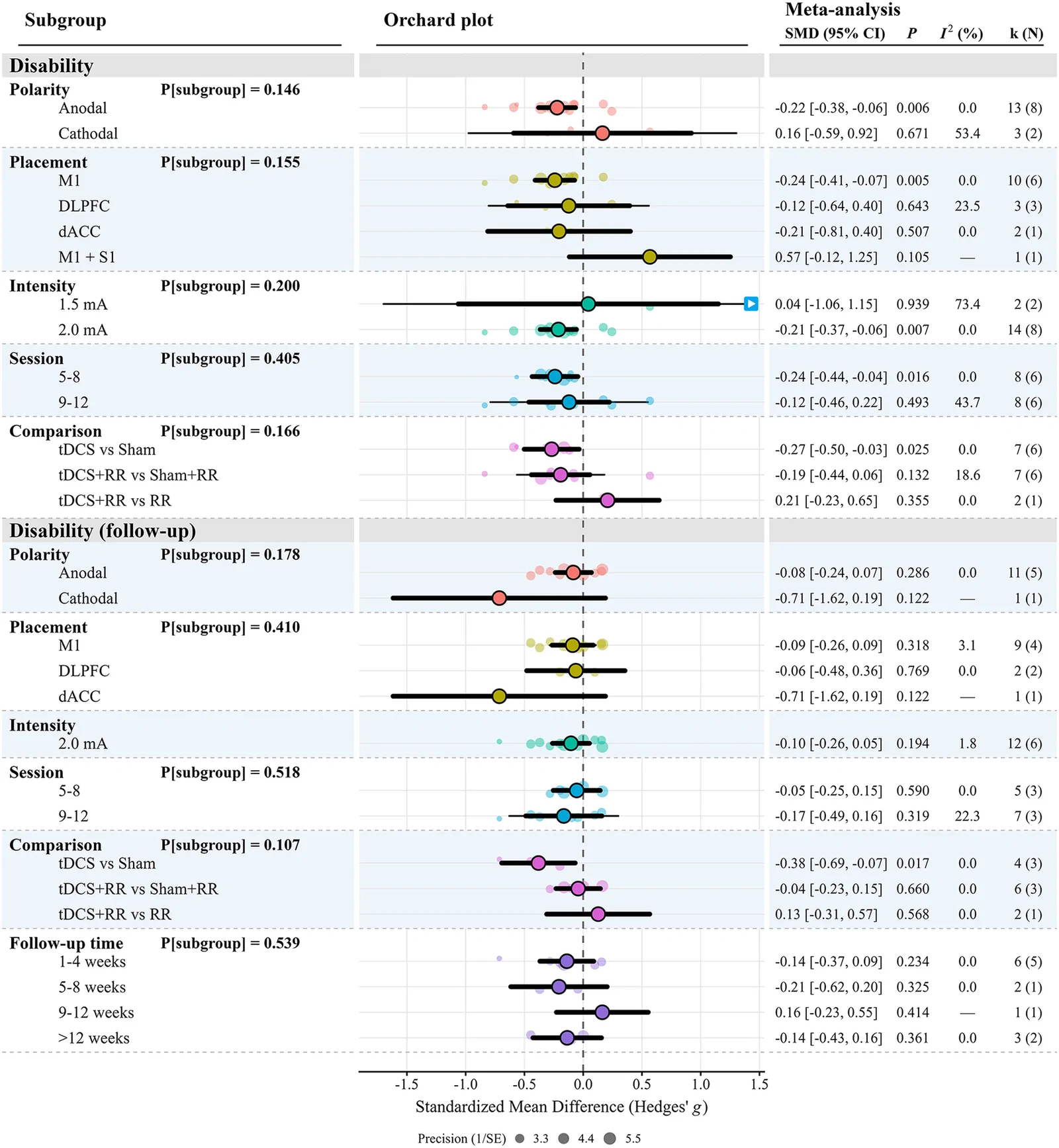
Subgroup analyses based on pain and follow-up pain. K, the total number of effects included in the pooled effect size; M1, primary motor cortex; DLPFC, dorsolateral prefrontal cortex; dACC, dorsal anterior cingulate cortex; S1, primary somatosensory cortex; tDCS, transcranial direct current stimulation; RR, routine rehabilitation.

#### 3.4.2 Follow-up pain

The follow-up pain outcomes incorporated 20 pairwise comparisons, and moderate heterogeneity was observed across studies (*I*^2^ = 44.0%). Meta-analysis results indicated that tDCS did not alleviate follow-up pain in individuals with CLBP compared with the control group (SMD = −0.19, 95% CI: −0.46–0.08, *P* = 0.173). Subgroup analyses showed variations in effect estimates according to number of treatment sessions and comparison type; however, no significant between-subgroup differences were detected (Figure 3; Supplementary Figure S3).

#### 3.4.3 Disability

Figure 4 and Supplementary Figure S3 illustrate the results of the meta-analysis on disability. This outcome measure included 16 pairwise comparisons, and low heterogeneity was found across studies (*I*^2^ = 2.2%). The meta-analysis results demonstrated that tDCS significantly reduced disability in individuals with CLBP compared with the control group (SMD = −0.18, 95% CI: −0.34–−0.03, *P* = 0.02). Numerical differences in effect estimates were observed across polarity, electrode placement, current intensity, number of treatment sessions, and comparison type, but these differences were not statistically significant between subgroups (Figure 4).

**Figure 4:**
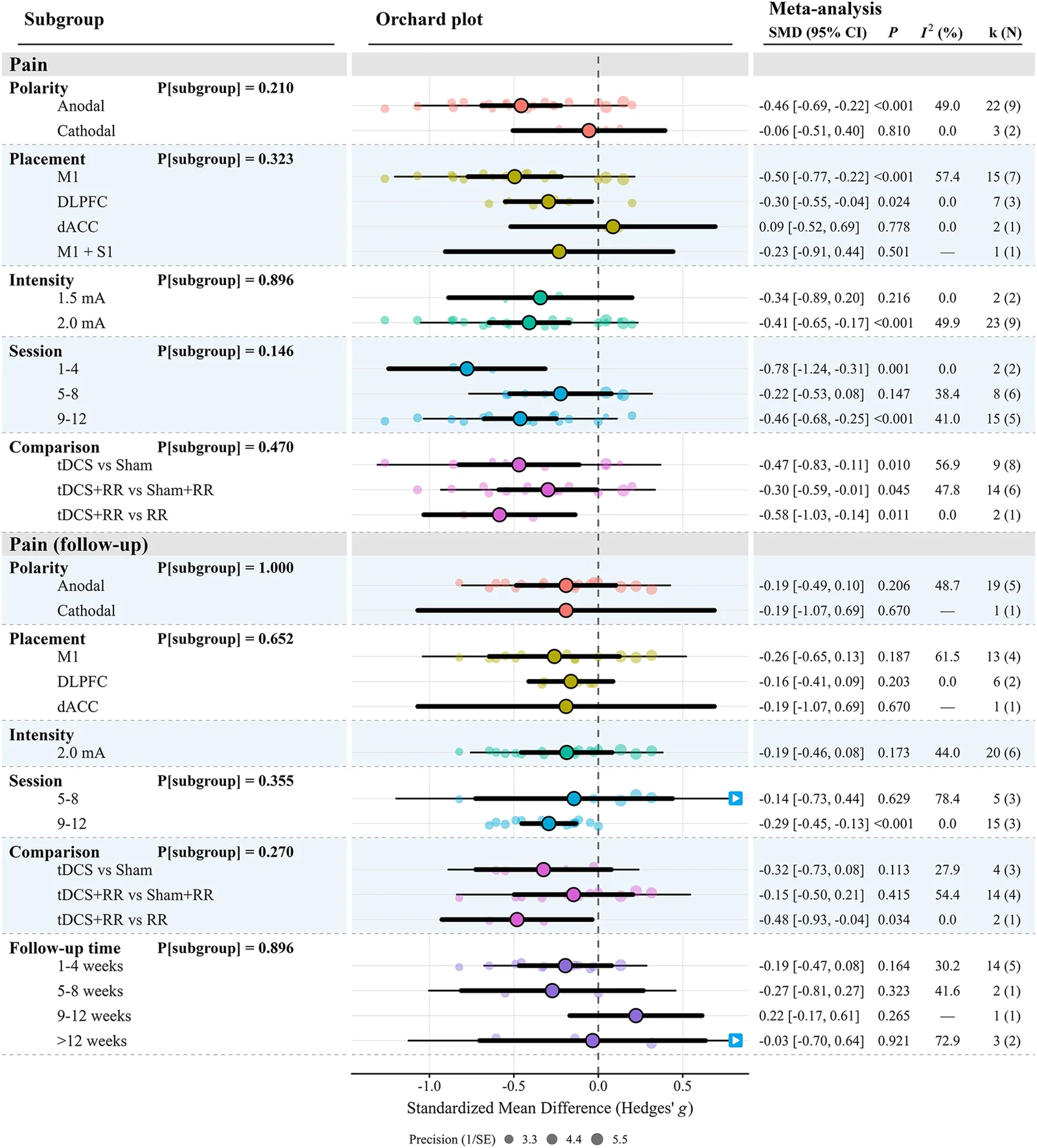
Subgroup analyses based on disability and follow-up disability. K, the total number of effects included in the pooled effect size; M1, primary motor cortex; DLPFC, dorsolateral prefrontal cortex; dACC, dorsal anterior cingulate cortex; S1, primary somatosensory cortex; tDCS, transcranial direct current stimulation; RR, routine rehabilitation.

#### 3.4.4 Follow-up disability

The follow-up disability outcomes included 12 pairwise comparisons, and low heterogeneity was found among studies (*I*^2^ = 1.8%). Meta-analysis revealed that tDCS did not reduce follow-up disability in individuals with CLBP (SMD = −0.10, 95% CI: −0.26–0.05, *P* = 0.194). Subgroup analyses showed variations in effect estimates across comparison types; however, no significant between-subgroup differences were detected (Figure 4; Supplementary Figure S3).

#### 3.4.5 Quality of life

Figure 5 and Supplementary Figure S3 illustrate the results of the meta-analysis for quality of life. This outcome measure included 25 pairwise comparisons, and moderate heterogeneity was observed across the studies (*I*^2^ = 47.2%). Meta-analysis results showed no significant difference in quality of life between the tDCS and control groups (SMD = 0.17, 95% CI: −0.08–0.41, *P* = 0.179). No significant between-subgroup differences were observed (Figure 5).

**Figure 5:**
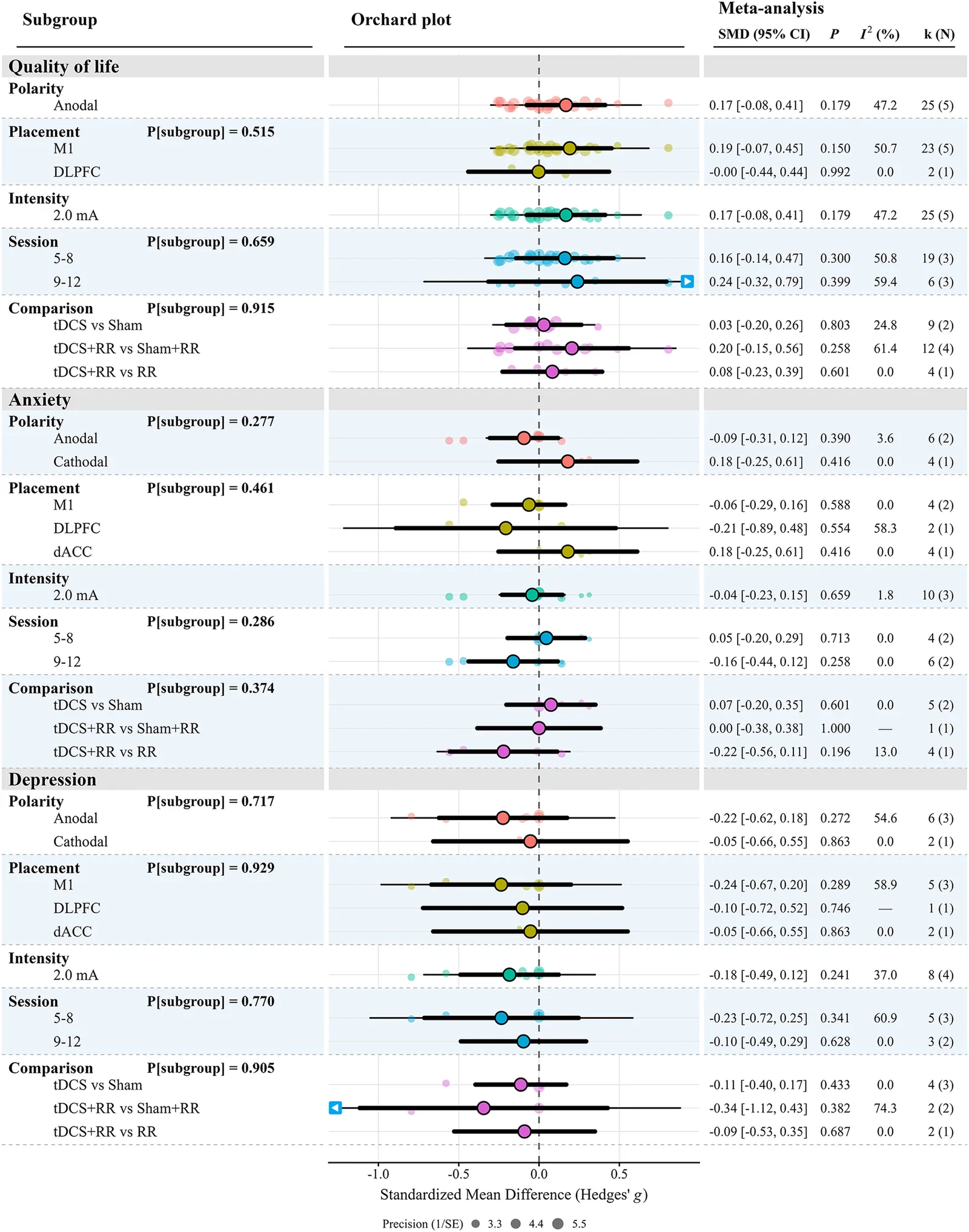
Subgroup analyses based on quality of life, anxiety, and depression. K, the total number of effects included in the pooled effect size; M1, primary motor cortex; DLPFC, dorsolateral prefrontal cortex; dACC, dorsal anterior cingulate cortex; tDCS, transcranial direct current stimulation; RR, routine rehabilitation.

#### 3.4.6 Anxiety

The anxiety outcomes included 10 pairwise comparisons, with low heterogeneity among the studies (*I*^2^ = 1.8%). Meta-analysis results showed that tDCS did not alleviate anxiety in individuals with CLBP compared with the control group (SMD = −0.04, 95% CI: −0.23–0.15, *P* = 0.659). Subgroup analysis revealed no significant differences between groups (Figure 5; Supplementary Figure S3).

#### 3.4.7 Depression

Figure 5 and Supplementary Figure S3 present the results of the meta-analysis for depression. This outcome measure included 8 pairwise comparisons, with moderate heterogeneity among the studies (*I*^2^ = 37.0%). Meta-analysis results showed that compared to the control group, tDCS did not alleviate depression in individuals with CLBP (SMD = −0.18, 95% CI: −0.49–0.12, *P* = 0.241). Subgroup analysis revealed no significant differences between groups (Figure 5).

### 3.5 Sensitivity analysis and publication bias

Supplementary Figure S4 presents a sensitivity analysis conducted using alternative correlation coefficients (*r* = 0.3 and *r* = 0.7). The overall effect estimates and their confidence intervals were generally similar to those obtained using the primary assumption of *r* = 0.5. When r was increased to 0.7, the effect estimate for disability was no longer statistically significant, whereas the significance of the remaining outcomes was unchanged.

Sensitivity analyses based on the leave-one-study-out approach demonstrated that the pooled effect estimates for pain, follow-up pain, follow-up disability, quality of life anxiety, and depression were stable. For disability, the direction of the effect estimates remained generally consistent, but statistical significance changed after the exclusion of individual studies, suggesting that the significance of the pooled effect was not fully robust (Supplementary Figure S5). Publication bias was assessed for pain and disability, with no significant evidence of publication bias observed for either outcome (*P* > 0.05, Supplementary Figure S6).

### 3.6 Meta-regression

Regression analysis results showed that the percentage of males was significantly associated with pain (*β* = 1.11, 95% CI: 0.02–2.21, *P* = 0.046, Supplementary Table S4).

### 3.7 Adverse events/side effects

Across the 14 studies evaluating adverse events or side effects, mild or transient effects, such as burning or heat sensations, difficulty concentrating, dizziness, drowsiness, headache, increased fatigue, mood changes, nausea, pain, skin redness, sleepiness, and tingling or itching at the electrode site, were commonly reported. Overall, the reported frequencies of these adverse events appeared comparable between the active and sham stimulation groups.

## 4 Discussion

This systematic review and meta-analysis of 14 RCTs suggests that tDCS may provide benefits for pain and disability in individuals with CLBP. The improvement in disability was small, and the statistical significance of this outcome was sensitive to the exclusion of individual studies. No clear overall benefits were observed for quality of life, anxiety, or depression. Overall, the certainty of evidence across the evaluated outcomes ranged from low to moderate. Subgroup analyses showed variation in effect estimates across different stimulation parameters and comparison types.

### 4.1 Main findings

The results of this study demonstrated that tDCS can significantly alleviate pain in individuals with CLBP. Although the mechanisms underlying the analgesic effects observed in the present meta-analysis were not directly examined, previous experimental and neurophysiological studies have proposed several potential mechanisms (52, 53). tDCS-induced increases in motor cortex excitability can modulate cortico–striatal–thalamic circuits and inhibit pain impulses originating from the spinal cord (54, 55). Furthermore, the neurophysiological effects of tDCS are not confined to the regions beneath the electrodes and can alter the structure of unstimulated brain regions and functional connectivity among brain regions (56). Given the complex neural pathways involved in pain processing that extend to distal brain regions, the distal effect of tDCS is considerable. The activation of thalamic nuclei may engage other pain-related structures (e.g., anterior cingulate cortex and periaqueductal gray matter) by releasing endogenous opioids (mu-opioid receptor mechanisms) (57, 58).

Apart from pain reduction, we found that tDCS reduced disability. Pain and disability are closely interrelated in individuals with CLBP, and greater pain has been associated with poorer physical function and greater psychological distress (59, 60). In a study investigating interventions for individuals with CLBP, a significant association was observed between reductions in pain intensity (VAS scores) and improvements in disability (ODI scores) within one week (61). These findings suggest that changes in pain and disability may be interrelated. However, the present meta-analysis did not evaluate their temporal or mediating relationship, and no causal pathway can therefore be inferred. Meanwhile, the functional improvements induced by tDCS may be related to plasticity modulation mechanisms in the central nervous system. Such modulation may affect neural networks governing motor planning, motor execution, and pain–emotion associations. Notably, brain plasticity can enhance motor control, thereby improving functional mobility (62).

Previous experimental and neurophysiological studies have suggested that anodal and cathodal tDCS may exert different effects on cortical excitability and pain processing (63, 64). Changes in cortical excitability induced by anodal cortical stimulation can effectively alleviate acute and chronic pain (63). A recent study indicates that anodal tDCS targeting the M1 area significantly reduces thermal pain, and cathodal tDCS increases thermal pain (64). The analgesic effect of anodal M1-tDCS may reflect reduced involvement of descending pain modulatory pathways and enhanced inhibitory control over pain-processing regions. Preclinical studies suggest that brainstem structures, such as the periaqueductal gray and rostral ventromedial medulla, are associated with pain modulation after M1 stimulation (65), while regulating thalamocortical circuitry (52, 66). This process may further influence pain perception. Conversely, cathodal tDCS may reduce cortical excitability and disrupt these pathways, thereby increasing pain perception (67). In the present study, anodal tDCS was associated with significant reductions in pain and disability, whereas cathodal tDCS was not. However, the differences between the two polarity subgroups were not statistically significant.

Multiple prior studies have confirmed that anodal tDCS increases motor-evoked potential amplitude and M1 excitability and enhanced M1 excitability correlates with pain modulation (66). The widespread electric field distribution of M1-tDCS modulates the activity of not only target areas but also the entire network of cortical regions structurally or functionally connected to the electrode placement (68). M1 serves as a critical pathway for the cortico–thalamic descending pathway, which plays an essential role in pain processing. Polania et al. (69) demonstrated that M1-tDCS enhanced functional connectivity between the motor cortex and thalamus and thereby reduced pain intensity. These findings from previous studies provide a potential mechanistic context for understanding the effects of M1 stimulation. In the present subgroup analyses, statistically significant reductions in pain and disability were observed in studies targeting M1. Notably, the evidence base was unevenly distributed across stimulation sites, with relatively few studies targeting cortical regions other than M1. Therefore, further research is needed to determine whether treatment effects differ across stimulation targets.

Subgroup analyses showed variation in effect estimates across different stimulation intensities. Previous experimental studies have suggested that stimulation intensity may influence the neurophysiological effects of tDCS (70, 71). Within established safety parameters, higher stimulation intensities may alter the magnitude and spatial distribution of the induced electric field, thereby affecting the neuronal populations engaged. Preclinical studies have reported intensity-dependent differences in neuromodulatory effects without a corresponding increase in tissue damage under the stimulation conditions examined (70). Neuroimaging and electrophysiological studies have indicated that 2 mA stimulation, compared with 1 mA or lower intensities, induces broader functional reorganization in cortico–cortical and corticospinal pathways, thus effectively activating the endogenous analgesic system (71). Notably, the subgroup findings in the present study were derived from comparisons across different studies, which may have differed in electrode placement, treatment sessions, co-interventions, and other methodological characteristics. Therefore, these findings should be interpreted with caution.

Subgroup analyses suggested that pain reductions were observed in studies using 9–12 treatment sessions, while improvements in disability were observed in studies using 5–8 sessions. However, no statistically significant between-subgroup differences were detected. Given that tDCS exert cumulative effects (72, 73), short courses of tDCS may be insufficient to elicit significant changes in pain and function. Multiple tDCS sessions are believed to induce long-term-potentiation-like effects, thereby producing more sustained analgesic effects. These mechanisms may provide a potential context for understanding why the number of treatment sessions might be relevant to treatment outcomes. Notably, the different patterns observed for pain and disability suggest that any potential relationship between the number of treatment sessions and treatment effects may not be straightforward or linear.

No significant effects of tDCS were observed on anxiety or depression. However, these findings should be interpreted cautiously because relatively few studies with small sample sizes contributed to these outcomes, and psychological measures varied across studies, potentially limiting statistical power and increasing heterogeneity. Neurophysiological mechanisms may also partly explain the limited effects observed. As a subthreshold neuromodulation approach, tDCS modulates neuronal excitability rather than directly inducing action potentials, and its effects may therefore depend on the alignment between stimulated brain regions and those engaged during behavioral tasks. This correspondence may not always be straightforward, as baseline task-related neural activity can influence the predominant neural regions recruited (74, 75).

### 4.2 Comparison with other studies

This systematic review comprehensively evaluates the effects of tDCS on pain, disability, and psychosocial outcomes in individuals with CLBP. A meta-analysis published in 2020 demonstrated that tDCS did not considerably improve pain and disability (20). In contrast, the present review included 14 RCTs, substantially expanding the available evidence base. The differences between the two reviews may therefore partly reflect the inclusion of additional trials and the resulting increase in statistical precision, as well as differences in the included studies, effect estimates, and analytical approaches. Overall, the current evidence suggests that tDCS may provide benefits for pain and disability in individuals with CLBP. To further explore potential sources of variation in treatment effects, we conducted subgroup analyses across relevant tDCS characteristics and exploratory univariable meta-regression analyses when a sufficient number of independent studies were available.

### 4.3 Study limitations

Several limitations of this study should be highlighted. First, different measurement instruments were used across studies for some outcomes, which may have introduced conceptual and measurement heterogeneity. Although standardized mean differences were used to account for differences in measurement scales, this approach does not ensure that the instruments assess sufficiently comparable underlying constructs. Second, owing to the limited number of available studies, neurophysiological measurements, such as cortical activation, brain functional networks, and corticomuscular coherence, which can further elucidate underlying mechanisms, were not included. Future research should identify optimal biomarkers to determine whether a treatment induces the desired neurophysiological effects and elucidate the mechanisms by which neuromodulation influences pain. Such information aids in distinguishing responders from nonresponders during a neuromodulation therapy. In addition, the limited number of studies within some outcomes and subgroups, imbalanced evidence across stimulation parameters, and uncertainty in publication-bias assessment may limit the robustness of the findings.

## 5 Conclusions

Current evidence suggests that tDCS may reduce pain and produce a small improvement in disability in individuals with CLBP. Exploratory analyses suggested that treatment effects may vary across stimulation parameters and comparison types. Given the low-to-moderate certainty of the available evidence, these findings should be interpreted cautiously. Further rigorous clinical trials are needed to clarify the clinical effects of tDCS on subjective and objective outcomes in individuals with CLBP and to investigate the neurophysiological mechanisms underlying its potential therapeutic effects.

## Data Availability

The original contributions presented in the study are included in the article/Supplementary Material, further inquiries can be directed to the corresponding author.
