# Supplementary material for: Effects of transcranial direct current stimulation on patient-reported outcomes in chronic low back pain: a systematic review and three-level meta-analysis
Source: Front Med (Lausanne). 2026 Oct 7;13:1950897. doi: 10.3389/fmed.2026.1950897 (PMC13683132; doi:10.3389/fmed.2026.1950897)
Supplement: Supplementary file 1 [file Datasheet1.pdf]

Supplementary date

Table S1: PRISMA Checklist 2
Table S2: Search strategies 6
Table S2-1 PubMed 6
Table S2-2 Cochrane Library 6
Table S2-3 EMBASE 6
Table S2-4 Web of Science 7
Table S2-5 EBSCO 7
Table S3: Participant and intervention characteristics of included studies 8
Table S4: GRADE Certainty of evidence 11
Table S5: Meta-regression 11
Figure S1: Risk of bias graph 11
Figure S2: Risk of bias summary 12
Figure S3: Forest plot 12
Figure S3-1 Forest plot of pain 12
Figure S3-2 Forest plot of follow-up pain 13
Figure S3-3 Forest plot of disability 13
Figure S3-4 Forest plot of follow-up disability 13
Figure S3-5 Forest plot of quality of life 14
Figure S3-6 Forest plot of anxiety 14
Figure S3-7 Forest plot of depression 14
Figure S4: Sensitivity analyses using alternative correlation coefficients 15
Figure S4-1 Sensitivity analysis of the meta-analysis results assuming r = 0.3 15
Figure S4-2 Sensitivity analysis of the meta-analysis results assuming r = 0.7 15
Figure S5: Sensitivity analyses 16
Figure S5-1 Sensitivity analysis for pain 16
Figure S5-2 Sensitivity analysis for follow-up pain 16
Figure S5-3 Sensitivity analysis for disability 17
Figure S5-4 Sensitivity analysis for follow-up disability 17
Figure S5-5 Sensitivity analysis for quality of life 18
Figure S5-6 Sensitivity analysis for anxiety 18
Figure S5-7 Sensitivity analysis for depression 18
Figure S6: Publication bias 19
References 19

Table S1: PRISMA Checklist

Section and Topic	Item #	Checklist item	Location where item is reported
**TITLE**			
Title	1	Identify the report as a systematic review.	Page 1
**ABSTRACT**			
Abstract	2	See the PRISMA 2020 for Abstracts checklist.	Page 1
**INTRODUCTION**			
Rationale	3	Describe the rationale for the review in the context of existing knowledge.	Page 2-3
Objectives	4	Provide an explicit statement of the objective(s) or question(s) the review addresses.	Page 3
**METHODS**			
Eligibility criteria	5	Specify the inclusion and exclusion criteria for the review and how studies were grouped for the syntheses.	Page 4-5
Information sources	6	Specify all databases, registers, websites, organisations, reference lists and other sources searched or consulted to identify studies. Specify the date when each source was last searched or consulted.	Page 4-5
Search strategy	7	Present the full search strategies for all databases, registers and websites, including any filters and limits used.	Page 4, Table S2-1 to Table S2-5
Selection process	8	Specify the methods used to decide whether a study met the inclusion criteria of the review, including how many reviewers screened each record and each report retrieved, whether they worked independently, and if applicable, details of automation tools used in the process.	Page 5-6
Data collection process	9	Specify the methods used to collect data from reports, including how many reviewers collected data from each report, whether they worked independently, any processes for obtaining or confirming data from study investigators, and if applicable, details of automation tools used in the process.	Page 5-6
Data items	10a	List and define all outcomes for which data were sought. Specify whether all results that were compatible with each outcome domain in each study were sought (e.g. for all measures, time points, analyses), and if not, the methods used to decide which results to collect.	Page 5-6
	10b	List and define all other variables for which data were sought (e.g. participant and intervention characteristics, funding sources). Describe any assumptions made about any missing or unclear information.	Page 5-6

Section and Topic	Item #	Checklist item	Location where item is reported
Study risk of bias assessment	11	Specify the methods used to assess risk of bias in the included studies, including details of the tool(s) used, how many reviewers assessed each study and whether they worked independently, and if applicable, details of automation tools used in the process.	Page 6
Effect measures	12	Specify for each outcome the effect measure(s) (e.g. risk ratio, mean difference) used in the synthesis or presentation of results.	Page 6
Synthesis methods	13a	Describe the processes used to decide which studies were eligible for each synthesis (e.g. tabulating the study intervention characteristics and comparing against the planned groups for each synthesis (item #5)).	Page 6-7
	13b	Describe any methods required to prepare the data for presentation or synthesis, such as handling of missing summary statistics, or data conversions.	Page 6-7
	13c	Describe any methods used to tabulate or visually display results of individual studies and syntheses.	Page 6-7
	13d	Describe any methods used to synthesize results and provide a rationale for the choice(s). If meta-analysis was performed, describe the model(s), method(s) to identify the presence and extent of statistical heterogeneity, and software package(s) used.	Page 6-7
	13e	Describe any methods used to explore possible causes of heterogeneity among study results (e.g. subgroup analysis, meta-regression).	Page 6-7
	13f	Describe any sensitivity analyses conducted to assess robustness of the synthesized results.	Page 6-7
Reporting bias assessment	14	Describe any methods used to assess risk of bias due to missing results in a synthesis (arising from reporting biases).	Page 6-7
Certainty assessment	15	Describe any methods used to assess certainty (or confidence) in the body of evidence for an outcome.	Page 6
**RESULTS**			
Study selection	16a	Describe the results of the search and selection process, from the number of records identified in the search to the number of studies included in the review, ideally using a flow diagram.	Page 8, Figure 1
	16b	Cite studies that might appear to meet the inclusion criteria, but which were excluded, and explain why they were excluded.	Page 8-9

Section and Topic	Item #	Checklist item	Location where item is reported
Study characteristics	17	Cite each included study and present its characteristics.	Page 8-9, Table S3
Risk of bias in studies	18	Present assessments of risk of bias for each included study.	Page 9, Figure S1, Figure S2
Results of individual studies	19	For all outcomes, present, for each study: (a) summary statistics for each group (where appropriate) and (b) an effect estimate and its precision (e.g. confidence/credible interval), ideally using structured tables or plots.	Page 9-12<br>Figure 2-5
Results of syntheses	20a	For each synthesis, briefly summarise the characteristics and risk of bias among contributing studies.	Page 9-12<br>Figure 2-5
	20b	Present results of all statistical syntheses conducted. If meta-analysis was done, present for each the summary estimate and its precision (e.g. confidence/credible interval) and measures of statistical heterogeneity. If comparing groups, describe the direction of the effect.	Page 9-12, Figure 2-5<br>Figure S3-1 to Figure S3-8
	20c	Present results of all investigations of possible causes of heterogeneity among study results.	Page 12, Figure S5-1 to Figure S5-2, Table S5
	20d	Present results of all sensitivity analyses conducted to assess the robustness of the synthesized results.	Page 12, Figure S5-1 to Figure S5-8
Reporting biases	21	Present assessments of risk of bias due to missing results (arising from reporting biases) for each synthesis assessed.	Figure S6-1 to Figure S6-2
Certainty of evidence	22	Present assessments of certainty (or confidence) in the body of evidence for each outcome assessed.	Page 9, Figure 1, Table S4
**DISCUSSION**			
Discussion	23a	Provide a general interpretation of the results in the context of other evidence.	Page 13-17
	23b	Discuss any limitations of the evidence included in the review.	Page 18
	23c	Discuss any limitations of the review processes used.	Page 18-19
	23d	Discuss implications of the results for practice, policy, and future research.	Page 17-18
**OTHER INFORMATION**			
Registration and protocol	24a	Provide registration information for the review, including register name and registration number, or state that the review was not registered.	Page 3-4
	24b	Indicate where the review protocol can be accessed, or state that a protocol was not prepared.	Page 3-4
	24c	Describe and explain any amendments to information	Page 3-4

Section and Topic	Item #	Checklist item	Location where item is reported
		provided at registration or in the protocol.	
Support	25	Describe sources of financial or non-financial support for the review, and the role of the funders or sponsors in the review.	The study was supported by *** and ***
Competing interests	26	Declare any competing interests of review authors.	Not report
Availability of data, code and other materials	27	Report which of the following are publicly available and where they can be found: template data collection forms; data extracted from included studies; data used for all analyses; analytic code; any other materials used in the review.	All datasets for this study are included in the manuscript and the Supplementary data

From: Page MJ, McKenzie JE, Bossuyt PM, Boutron I, Hoffmann TC, Mulrow CD, et al. The PRISMA 2020 statement: an updated guideline for reporting systematic reviews. BMJ 2021;372:n71. doi: 10.1136/bmj.n71. This work is licensed under CC BY 4.0. To view a copy of this license, visit https://creativecommons.org/licenses/by/4.0/

Table S2: Search strategies

Table S2-1 PubMed

Concept	Query
#1	("Back Pain"[Mesh]) OR ("Low Back Pain"[Mesh]) OR (back pain[Title/Abstract]) OR (low back pain[Title/Abstract]) OR (back pain, low[Title/Abstract]) OR (back pains, low[Title/Abstract]) OR (low back pains[Title/Abstract]) OR (pain, low back[Title/Abstract]) OR (pains, low back[Title/Abstract]) OR (low back ache[Title/Abstract]) OR (ache, low back[Title/Abstract]) OR (low-back pain[Title/Abstract]) OR (chronic low back pain[Title/Abstract]) OR (chronic low-back pain[Title/Abstract]) OR (CLBP[Title/Abstract])OR (lumbago[Title/Abstract])
#2	("transcranial direct current stimulation"[Mesh]) OR (transcranial direct current stimulation[Title/Abstract]) OR (tDCS[Title/Abstract]) OR (anodal stimulation transcranial direct current stimulation[Title/Abstract]) OR (anodal stimulation tDCS[Title/Abstract]) OR (cathodal stimulation transcranial direct current stimulation[Title/Abstract]) OR (cathodal stimulation tDCS[Title/Abstract]) OR (transcranial electrical stimulation[Title/Abstract]) OR (non-invasive brain stimulation[Title/Abstract])
#3	#1 AND #2

Table S2-2 Cochrane Library

Concept	Query
#1	("Back Pain"[Mesh]) OR ("Low Back Pain"[Mesh])
#2	Title, abstract, and keywords = (back pain OR low back pain OR back pain, low OR back pains, low OR low back pains OR pain, low back OR pains, low back OR low back ache OR ache, low back OR low-back pain OR chronic low back pain OR chronic low-back pain OR CLBP OR lumbago)
#3	#1 OR #2
#4	("transcranial direct current stimulation"[Mesh]) OR ("tDCS"[Mesh])
#5	Title, abstract, and keywords = (back pain OR low back pain OR back pain, low OR back pains, low OR low back pains OR pain, low back OR pains, low back OR low back ache OR ache, low back OR low-back pain OR chronic low back pain OR chronic low-back pain OR CLBP OR lumbago)
#5	#4 OR #5
#6	#3 AND #5

Table S2-3 EMBASE

Concept	Query
#1	'back pain'/exp OR 'back pain' OR (('back'/exp OR back) AND ('pain'/exp OR pain)) OR 'low back pain'/exp OR 'low back pain' OR (low AND ('back'/exp OR back) AND ('pain'/exp OR pain)) OR 'back pain, low'/exp OR 'back pain, low' OR (('back'/exp OR back) AND ('pain,'/exp OR pain,) AND low) OR 'back pains, low' OR (('back'/exp OR back) AND pains, AND low) OR 'low back pains' OR (low AND ('back'/exp OR back) AND pains) OR 'pain, low back'/exp OR 'pain, low back' OR (('pain,'/exp OR pain,) AND low AND ('back'/exp OR back)) OR 'pains, low back' OR (pains, AND

	low AND ('back'/exp OR back)) OR 'low back ache' OR (low AND ('back'/exp OR back) AND ache) OR 'ache, low back' OR (ache, AND low AND ('back'/exp OR back)) OR 'low-back pain'/exp OR 'low-back pain' OR ('low back' AND ('pain'/exp OR pain)) OR 'chronic low back pain'/exp OR 'chronic low back pain' OR (chronic AND low AND ('back'/exp OR back) AND ('pain'/exp OR pain)) OR 'chronic low-back pain'/exp OR 'chronic low-back pain' OR (chronic AND 'low back' AND ('pain'/exp OR pain)) OR clbp OR 'lumbago'/exp OR lumbago
#2	'transcranial direct current stimulation'/exp OR 'transcranial direct current stimulation' OR (transcranial AND ('direct'/exp OR direct) AND current AND ('stimulation'/exp OR stimulation)) OR tdcs OR 'anodal stimulation transcranial direct current stimulation' OR (anodal AND transcranial AND ('direct'/exp OR direct) AND current AND ('stimulation'/exp OR stimulation)) OR 'anodal stimulation tdcs' OR (anodal AND ('stimulation'/exp OR stimulation) AND tdcs) OR 'cathodal stimulation transcranial direct current stimulation' OR (cathodal AND transcranial AND ('direct'/exp OR direct) AND current AND ('stimulation'/exp OR stimulation)) OR 'cathodal stimulation tdcs' OR (cathodal AND ('stimulation'/exp OR stimulation) AND tdcs) OR 'transcranial electrical stimulation'/exp OR 'transcranial electrical stimulation' OR (transcranial AND electrical AND ('stimulation'/exp OR stimulation)) OR 'non-invasive brain stimulation' OR ('non invasive' AND ('brain'/exp OR brain) AND ('stimulation'/exp OR stimulation))
#3	#1 AND #2

Table S2-4 Web of Science

Concept	Query
#1	Topic, title, and abstract = (back pain OR low back pain OR back pain, low OR back pains, low OR low back pains OR pain, low back OR pains, low back OR low back ache OR ache, low back OR low-back pain OR chronic low back pain OR chronic low-back pain OR CLBP OR lumbago)
#2	Topic, title, and abstract = (transcranial direct current stimulation OR tDCS OR anodal stimulation transcranial direct current stimulation OR anodal stimulation tDCS OR cathodal stimulation transcranial direct current stimulation OR cathodal stimulation tDCS OR transcranial electrical stimulation OR non-invasive brain stimulation)
#3	#1 AND #2

Table S2-5 EBSCO

Concept	Query
#1	Subject terms, title, and abstract = (back pain OR low back pain OR back pain, low OR back pains, low OR low back pains OR pain, low back OR pains, low back OR low back ache OR ache, low back OR low-back pain OR chronic low back pain OR chronic low-back pain OR CLBP OR lumbago)
#2	Subject terms, title, and abstract = (transcranial direct current stimulation OR tDCS OR anodal stimulation transcranial direct current stimulation OR anodal stimulation tDCS OR cathodal stimulation transcranial direct current stimulation OR cathodal stimulation tDCS OR transcranial electrical stimulation OR non-invasive brain stimulation)
#3	#1 AND #2

Table S3: Participant and intervention characteristics of included studies

Author, Year	Country	Number of participants	Age (years)	Pain duration (months)	tDCS/other intervention	Control/ comparators	Outcome
Alcon 2025[1]	United States	10 (10F, 0M)/ 10 (8F, 2M)	46.8±15.4/ 35.9±17.4	71.6±91.3/ 52.4±43.2	Anodal-tDCS, tDCS parameters:<br>Electrode = $25cm^2$, Placement = DLPFC<br>Intensity = 2.0 mA, Density = 0.08 mA/cm^2<br>Duration = 20 min, Session = 5 (2 weeks)	Sham-tDCS	**Pain:** NPRS<br>**Disability:** MODI<br>Outcomes were assessed before and after intervention
Armbrust 2024[2]	Brazil	24 (15F, 9M)/ 24 (18F, 6M)	38.3±12.8/ 38.8±15.3	More than 3 months	Anodal-tDCS + osteopathic manipulative treatment, tDCS parameters:<br>Electrode = $25cm^2$, Placement = M1<br>Intensity = 2.0 mA, Density = 0.08 mA/cm^2<br>Duration = 20 min, Session = 10 (2 weeks)	Sham tDCS + osteopathic manipulative treatment	**Pain:** VAS<br>**Disability:** RMDQ<br>**Quality of life:** EQ-5D-3-Level<br>Outcomes were assessed at before and after intervention and at 1 month follow-up
Corti 2022[3]	Australia	19 (9F, 10M)	49.7±14.3/ 60.7±12.8	147.6±111.6/ 188.4±202.8	Anodal-tDCS, tDCS parameters:<br>Electrode = 5*7 cm ($35cm^2$), Placement = DLPFC<br>Intensity = 1.5 mA, Density = 0.043 mA/cm^2<br>Duration = 20 min, Session = 8 (4 weeks)	Sham-tDCS	**Pain:** VAS<br>**Disability:** RMDQ<br>Outcomes were assessed before and after intervention
Diniz 2026[4]	Brazil	20/<br>20<br>20	Not reported	More than 6 months	Anodal-tDCS + TENS, tDCS parameters:<br>Electrode = 4*4 cm ($16cm^2$), Placement = **a:** M1 or **b:** DLPFC<br>Intensity = 2.0 mA, Density = 0.125 mA/cm^2<br>Duration = 20 min, Session = 10 (2 weeks)	TENS	**Pain:** NPRS and BPI<br>**Disability:** RMDQ<br>**Quality of life:** SF-36 (physical and mental)<br>**Anxiety:** STAI (state and trait)<br>**Depression**: BDI<br>Outcomes were assessed before and after intervention and at 1 month follow-up
Hazime 2017[5]	Brazil	23 (19F, 4M)/ 23 (17F, 6M)/ 23 (18F, 5M)/ 23 (15F, 8M)	51.9±9.9/ 54.1±9.8/ 51.3±9.9/ 53.0±9.9	91.6±108.3/ 69.2±92.7/ 37.3±39.4/ 59.7±59.7	**a:** Anodal-tDCS + sham PES, **b:** Anodal-tDCS + PES, tDCS parameters:<br>Electrode = 5*7 cm ($35cm^2$), Placement: M1<br>Intensity = 2.0 mA, Density = 0.057 mA/cm^2<br>Duration = 20 min, Session = 12 (4 weeks)	**a:** Sham anodal-tDCS + sham PES<br>**b:** Sham anodal tDCS + PES	**Pain:** NPRS<br>**Disability:** RMDQ<br>Outcomes were assessed before and after intervention, and at 2 months, and 5 months follow-up

Jiang 2020[6]	China	26 (12F, 14M)/ 25 (12F, 13M)	39.9±14.2/ 44.1±13.0	25.6±48.9/ 29.5±46.2	Anodal-tDCS, tDCS Parameters:<br>Electrode = 0.78 cm^2, Placement = M1<br>Intensity = 2.0 mA, Density = 2.54 mA/cm^2<br>Duration = 20 min, Session = 1	Sham-tDCS	**Pain:** NPRS<br>Outcomes were assessed before and after intervention
Li 2026[7]	China	13 (6F, 7M)/ 13 (7F, 6M)	27.2±5.3/ 27.9±6.3	More than 3 months	Anodal-tDCS, tDCS Parameters:<br>Electrode = 5*7 cm (35cm^2), Placement = M1<br>Intensity = 2.0 mA, Density = 0.057 mA/cm^2<br>Duration = 20 min, Session = 1	Sham-tDCS	**Pain:** VAS<br>Outcomes were assessed before and after intervention
Luedtke 2015[9]	Germany	67 (33F, 34M)/ 68 (30F, 38M)	45.0±9.0/ 44.0±10.0	98±106/ 93±125	**Phase a:** Anodal-tDCS, **Phase b:** Anodal-tDCS + CBT, tDCS Parameters:<br>Electrode = 5*7 cm (35cm^2), Placement: M1<br>Intensity = 2.0 mA, Density = 0.057 mA/cm^2<br>Duration = 20 min, Session = 5 (1 week)	**Phase a:** Sham-tDCS<br>**Phase b:** Sham-tDCS + CBT	**Pain:** VAS<br>**Disability:** ODI<br>**Quality of life:** RAND-36<br>**Anxiety:** HADS anxiety<br>**Depression**: HADS depression<br>Outcomes were assessed before and after intervention and at 4 weeks, 12 weeks and 24 weeks follow-up
Mariano 2019[10]	United States	10 (1F, 9M)/ 11 (2F, 9M)	65.7±8.8/ 60.7±11.8	More than 6 months	Cathodal-tDCS, tDCS Parameters:<br>Electrode = 5*7 cm (35cm^2), Placement: dACC<br>Intensity = 2.0 mA, Density = 0.057 mA/cm^2<br>Duration = 20 min, Session = 10 (2 weeks)	Sham-tDCS	**Pain:** DVPRS<br>**Disability:** RMDQ<br>**Anxiety:** PASS, GAD-7<br>**Depression**: PHQ-9<br>Outcomes were assessed before and after intervention and at 4 weeks follow-up
Qanbari 2024[11]	Iran	17 (6F, 11M)/ 17 (10F, 7M)	35.7±8.5/ 42.5±11.4	12.0±25.9/ 36.0±57.8	Cathodal-tDCS+ sensory-motor exercises, tDCS Parameters:<br>Electrode = 2*4 cm (8cm^2), Placement: M1 + S1<br>Technique: 10 s ramp up/ramp down<br>Intensity = 1.5 mA, Density = 0.188 mA/cm^2<br>Duration = 20 min, Session = 12 (4 weeks)	Sham-tCDS + sensory-motor exercises	**Pain:** VAS<br>**Disability:** ODI<br>Outcomes were assessed before and after intervention

Shiasy 2020[12]	Iran	15/ 15	35.6±8.8/ 35.6±12.4	More than 3 months	Anodal-tDCS + attention bias modification, tDCS parameters:<br>Electrode = 4*4 cm (16cm^2), Placement: M1<br>Intensity = 2.0 mA, Density = 0.125 mA/cm^2<br>Duration = 20 min, Session = 5 (1 week)	Attention bias modification	**Pain:** BPI<br>**Disability:** RMDQ<br>**Anxiety:** DASS-Anxiety<br>**Depression**: DASS-Depression<br>Outcomes were assessed before and after intervention and at 1 month follow-up
Sornkaew 2024[13]	Thailand	12 (7F, 5M)/ 10 (5F, 5M)	29.2±7.0/ 29.8±8.0	43.6±47.0/ 35.6±53.8	Anodal-tDCS + motor control exercise, tDCS parameters:<br>Electrode = 5*7 cm (35cm^2), Placement: M1<br>Intensity = 2.0 mA, Density = 0.057 mA/cm^2<br>Duration = 20 min, Session = 12 (6 weeks)	Sham -tDCS + motor control exercise	**Disability:** MODI<br>**Quality of life:** SF-36<br>Outcomes were assessed at before and after intervention
Straudi 2018[14]	Italy	18 (12F, 6M)/ 17 (14F, 3M)	54.3±12.4/ 56.0±12.9	112.8±110.4/ 93.6±63.6	**Phase a:** Anodal-tDCS, **Phase b:** Anodal-tDCS + group exercise, tDCS parameters:<br>Electrode = 5*7 cm (35cm^2), Placement: M1<br>Intensity = 2.0 mA, Density = 0.057 mA/cm^2<br>Duration = 20 min, Session = 5 (1 week)	**Phase a:** Sham-tCDS<br>**Phase b:** Sham-tCDS + group exercise	**Pain:** VAS<br>**Disability:** RMDQ<br>**Quality of life:** EQ-5D-5-Level<br>**Depression**: PHQ-9<br>Outcomes were evaluated before and after intervention and at 1 month follow-up
Loan 2022[8]	Thailand	10 (4F, 6M)/ 10 (6F, 4M)	50.1±6.5/ 45.3±10.1	More than 6 months	Anodal-tDCS + physical therapy, tDCS parameters:<br>Electrode = 5*7 cm (35cm^2), Placement: M1<br>Intensity = 2.0 mA, Density = 0.057 mA/cm^2<br>Duration = 20 min, Session = 12 (4 weeks)	Physical therapy	**Pain:** VAS<br>**Quality of life:** WHOQOL<br>Outcomes were assessed before and after intervention and at 1 week follow-up

BDI: Back Depression Inventory; BPI: Brief Pain Inventory; CBT: cognitive behavioral therapy; dACC: dorsal anterior cingulate cortex; DASS: Depression Anxiety and Stress Scales; DVPRS: Defense and Veterans Pain Rating Scale; DLPFC: dorsolateral prefrontal cortex; EQ-5D: EuroQuol-5 Dimension; F: female; GAD-7: 7-item Generalized Anxiety Disorder scale; HADS: Hospital Anxiety and Depression Scale; M:Male; M1: primary motor cortex; NPRS: Numerical Pain Rating Scale; ODI: Oswestry Disability Index; PASS: Pain Anxiety Symptoms Scale: PCS: Pain Catastrophizing Scale; PES: Peripheral Electrical Stimulation; PHQ-9: 9-item Patient Health Questionnaire; QOL: quality of life; RMDQ: Roland-Morris Disability Questionnaire; S1: primary somatosensory cortex; SF-36: 36-item Short Form Health Survey; STAI: Spielberger State and Trait Anxiety Inventory; tDCS: transcranial direct electrical stimulation; TENS: Transcutaneous Electrical Nerve Stimulation; VAS: Visual Analogue Scale; WHOQOL: World Health Organization Quality of Life

Table S4: GRADE Certainty of evidence

Outcome	Risk of bias	Inconsistency	Indirectness	Imprecision	Publication bias	Certainty of evidence
Pain	Not serious	Serious	Not serious	Not serious	Not serious	Moderate
Follow-up pain	Not serious	Serious	Not serious	Serious	NA	Low
Disability	Not serious	Not serious	Not serious	Serious	Not serious	Moderate
Follow-up disability	Not serious	Not serious	Not serious	Serious	NA	Moderate
Quality of life	Not serious	Serious	Not serious	Serious	NA	Low
Anxiety	Not serious	Not serious	Not serious	Serious	NA	Moderate
Depression	Not serious	Serious	Not serious	Serious	NA	Low

GRADE: Grading of Recommendations Assessment, Development and Evaluation

Table S5: Meta-regression

Covariate	β (95% Confidence Interval)	Standard Error	*P*-value
**Pain**			
Mean age	0.02 (−0.01, 0.04)	0.01	0.275
Percentage of males	1.11 (0.02, 2.21)	0.56	**0.046**
Region	—	—	0.163
Publication year	−0.04 (−0.09, 0.01)	0.03	0.132
Outcome measures	—	—	0.466
**Disability**			
Mean age	0.00 (−0.02, 0.02)	0.01	0.953
Percentage of males	0.18 (−0.83, 1.18)	0.51	0.731
Region	—	—	0.909
Publication year	0.02 (−0.01, 0.06)	0.02	0.212
Outcome measures	—	—	0.406

*Significant influence factors, 95% CI does not contain zero; SEBT: Star excursion balance test; CAIT: Cumberland ankle instability tool; FAAM: Foot and ankle ability measure; ROB 2: Risk of Bias 2

Figure S1: Risk of bias graph

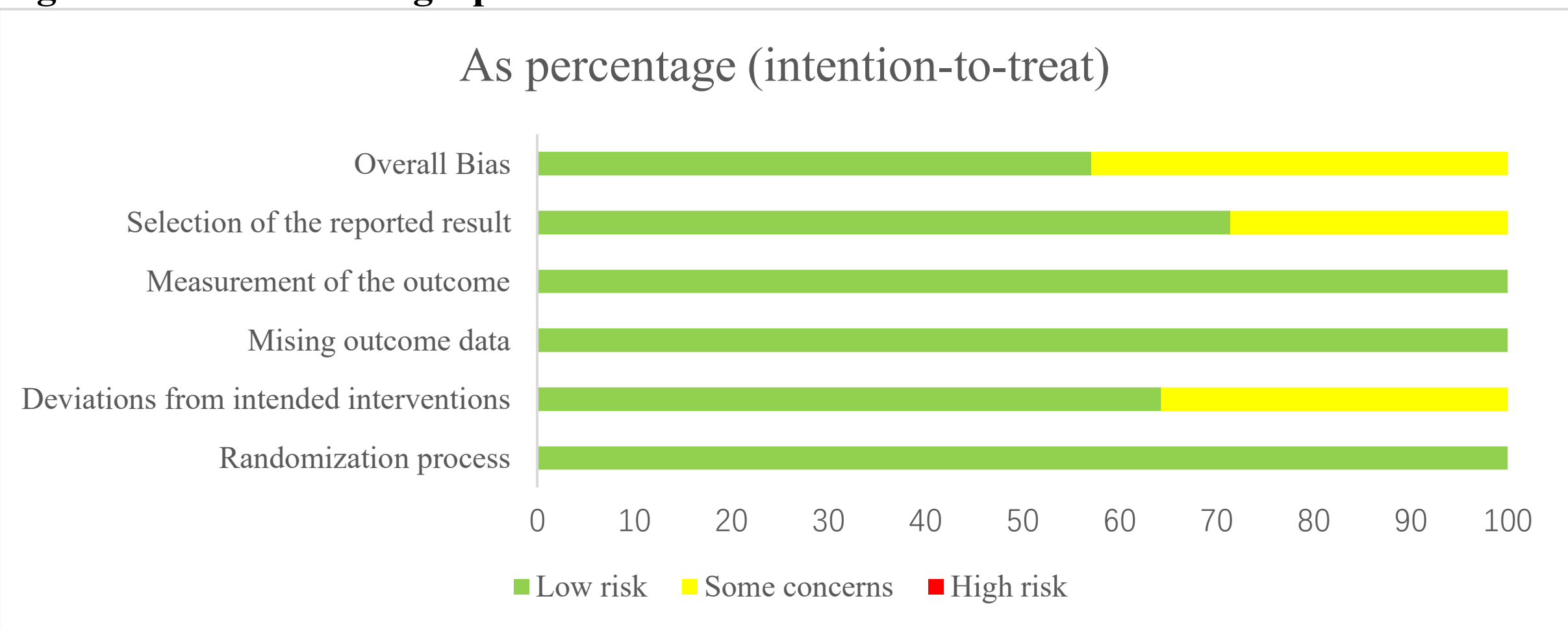

Figure S2: Risk of bias summary

Study ID	D1	D2	D3	D4	D5	Overall
Alcon 2025	+	+	+	+	+	+
Armbrust 2024	+	+	+	+	+	+
Corti 2022	+	!	+	+	+	!
Diniz 2026	+	+	+	+	!	!
Hazime 2017	+	+	+	+	+	+
Jiang 2020	+	+	+	+	+	+
Li 2026	+	!	+	+	+	!
Luedtke 2015	+	+	+	+	+	+
Mariano 2019	+	+	+	+	+	+
Qanbari 2024	+	+	+	+	+	+
Shiasy 2020	+	!	+	+	!	!
Sornkaew 2024	+	!	+	+	!	!
Straudi 2018	+	+	+	+	+	+
Loan 2022	+	!	+	+	!	!

+ Low risk
! Some concerns
- High risk

D1 Randomisation process
D2 Deviations from the intended interventions
D3 Missing outcome data
D4 Measurement of the outcome
D5 Selection of the reported result

Figure S3: Forest plot

Figure S3-1 Forest plot of pain

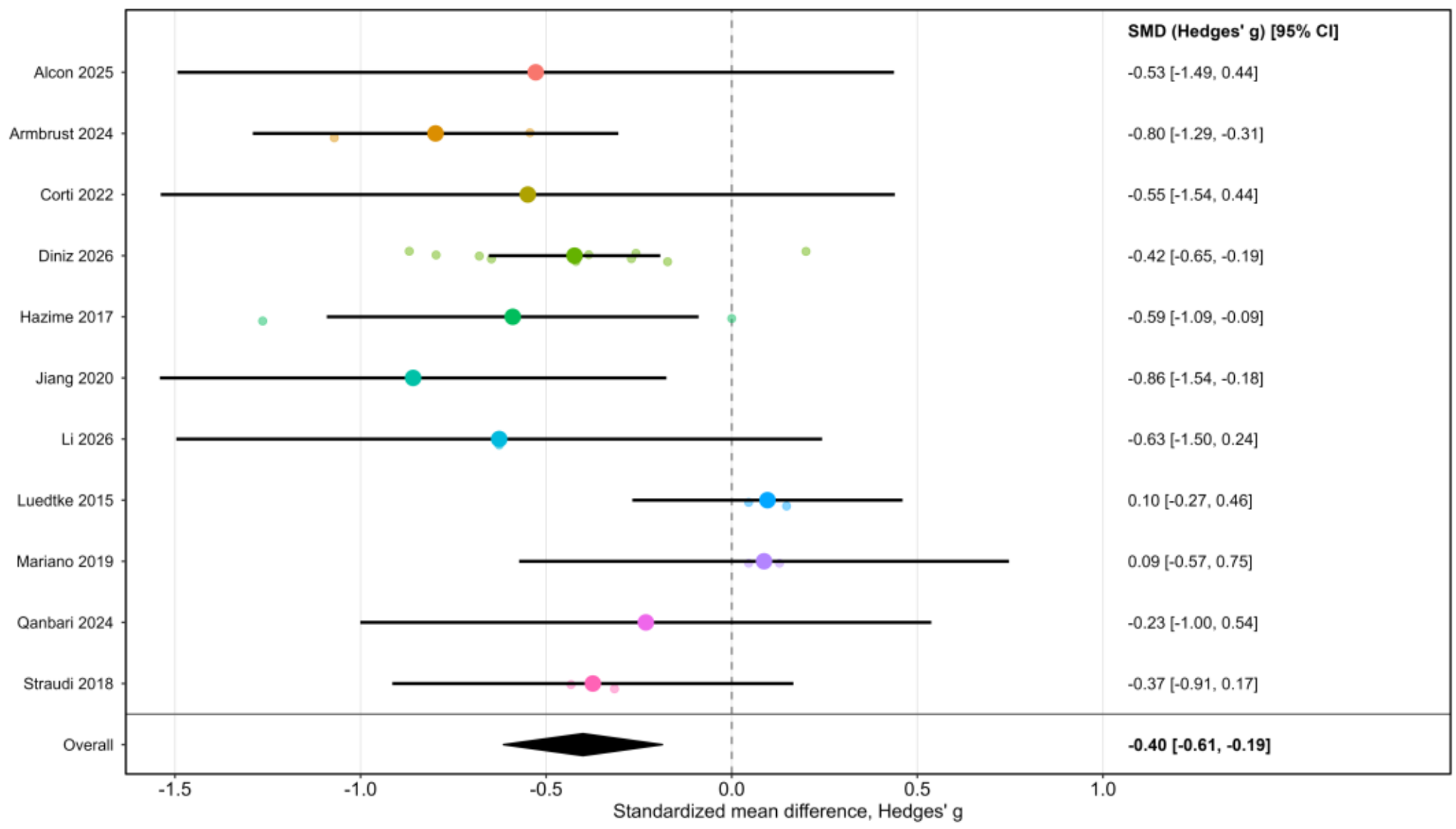

Figure S3-2 Forest plot of follow-up pain

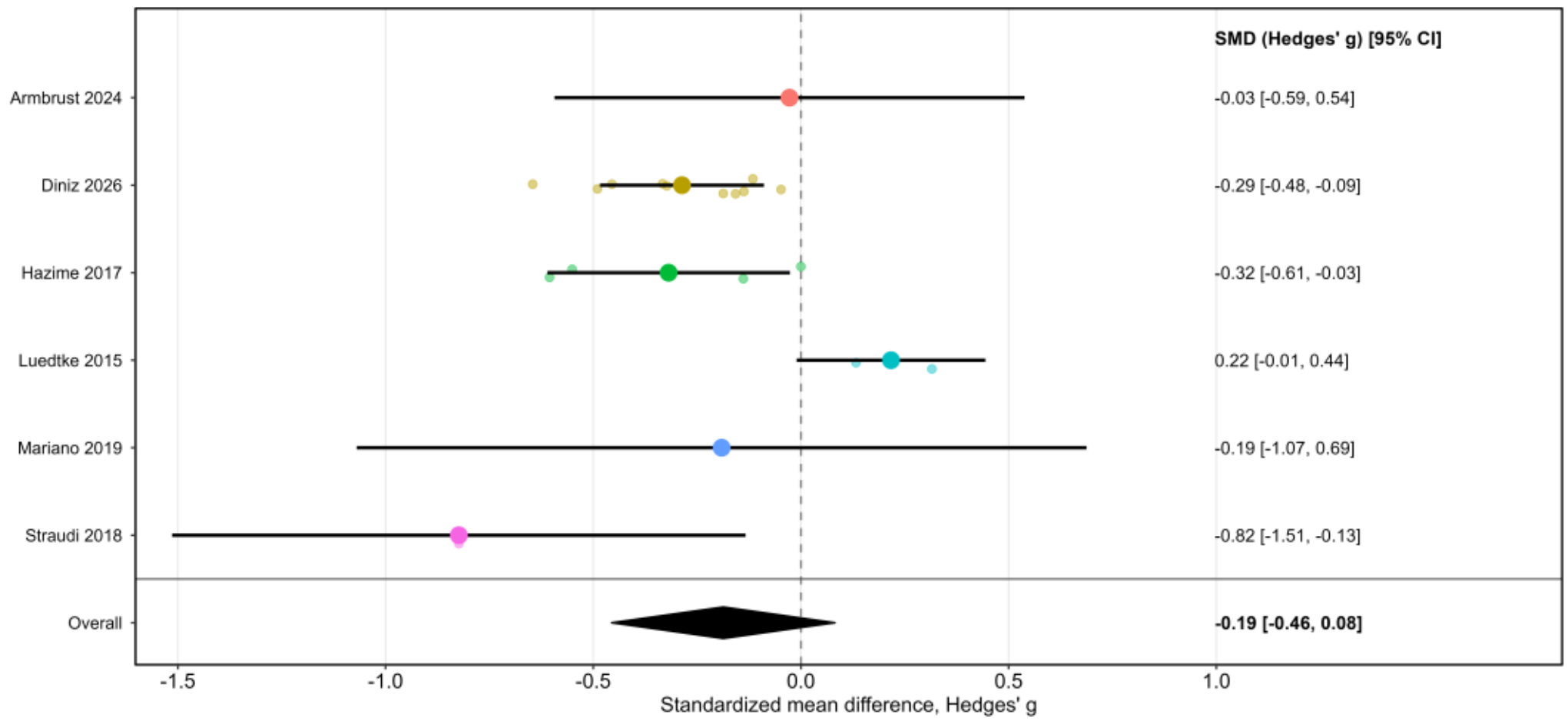

Figure S3-3 Forest plot of disability

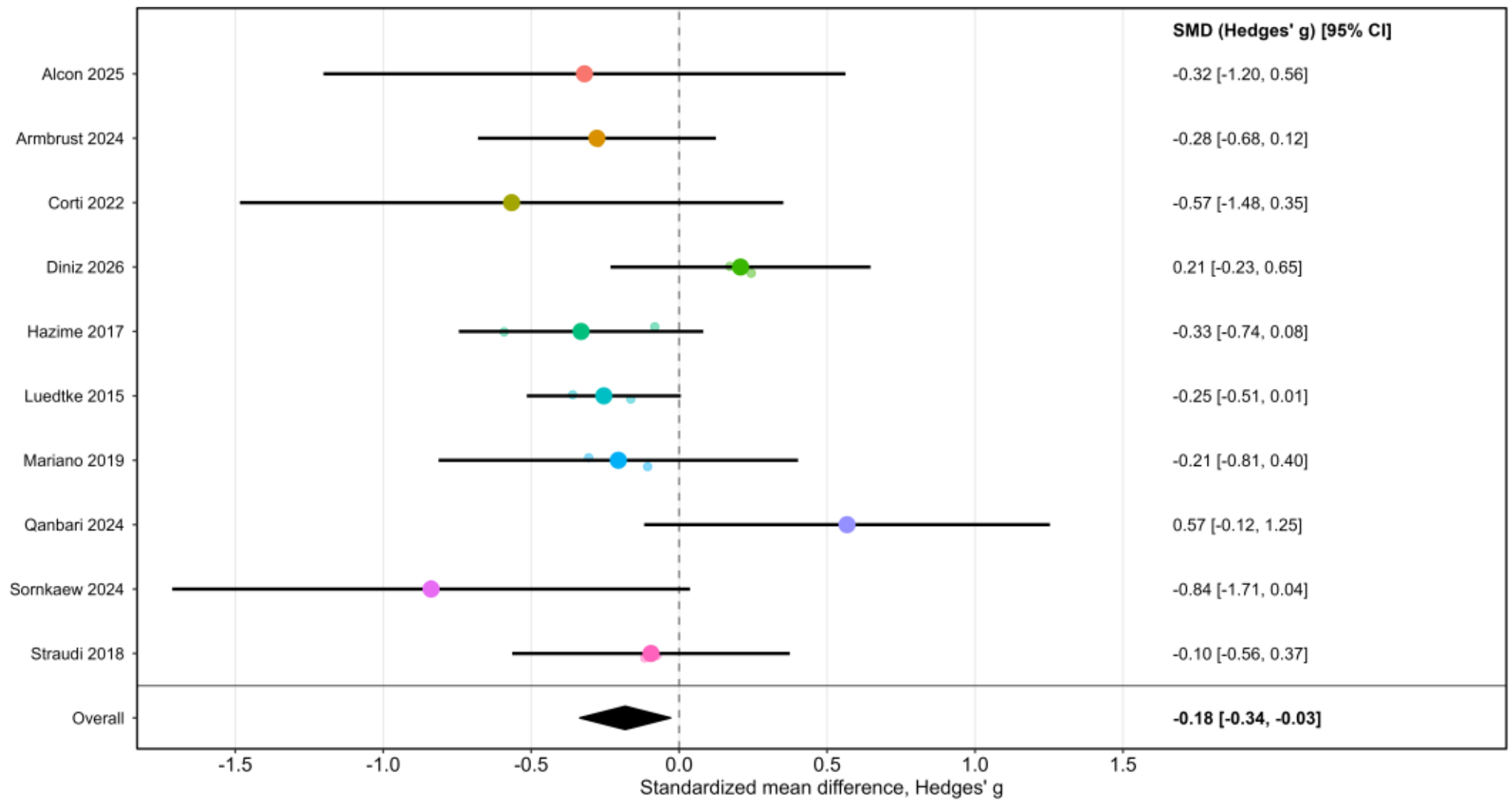

Figure S3-4 Forest plot of follow-up disability

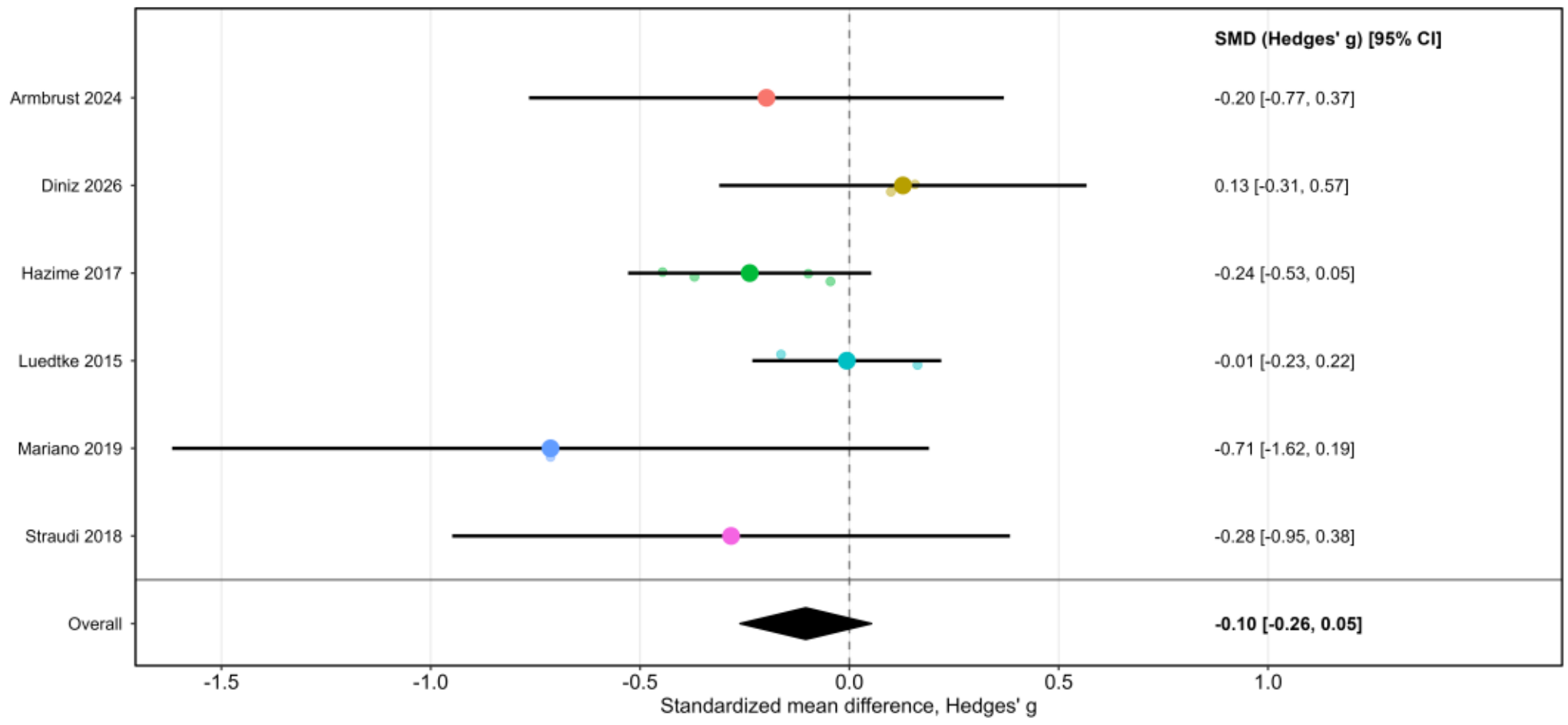

Figure S3-5 Forest plot of quality of life

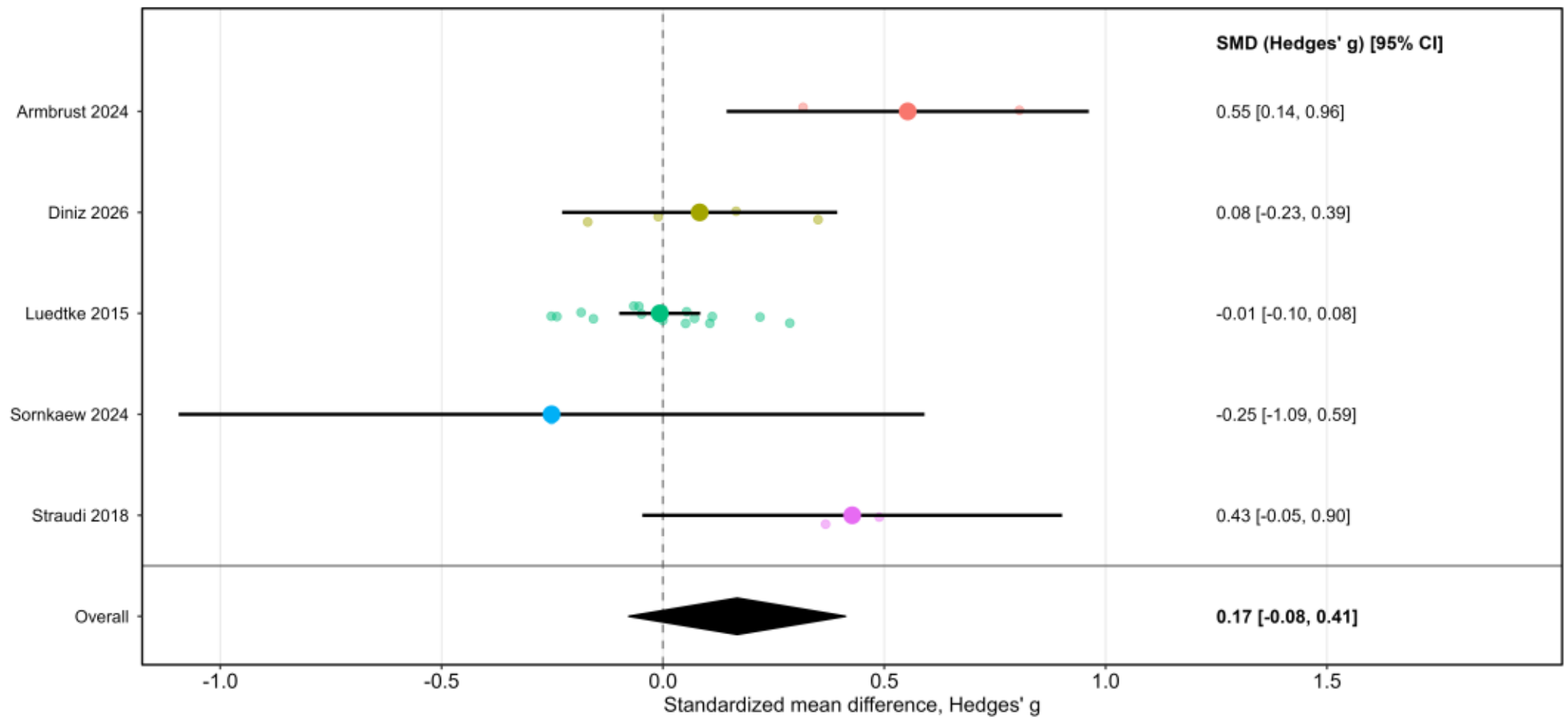

Figure S3-6 Forest plot of anxiety

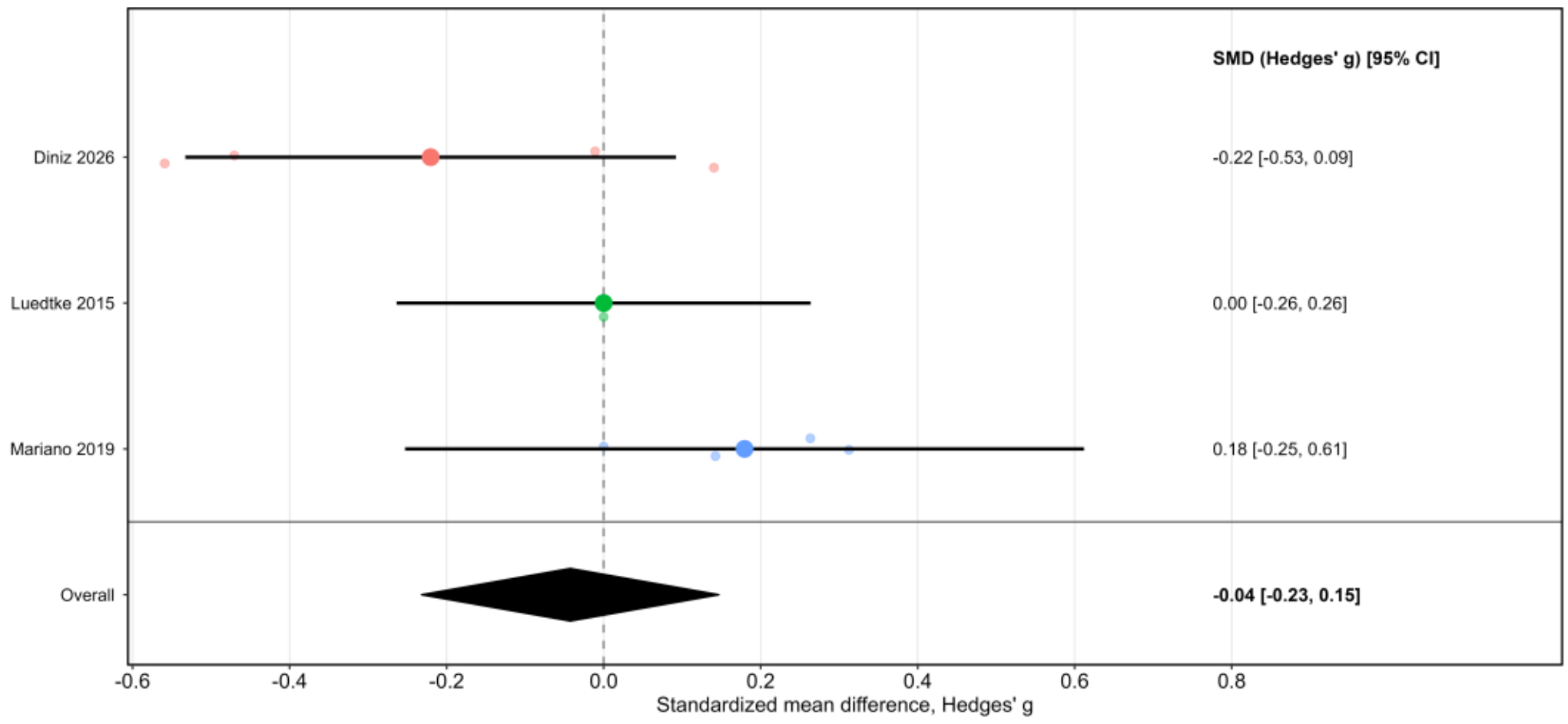

Figure S3-7 Forest plot of depression

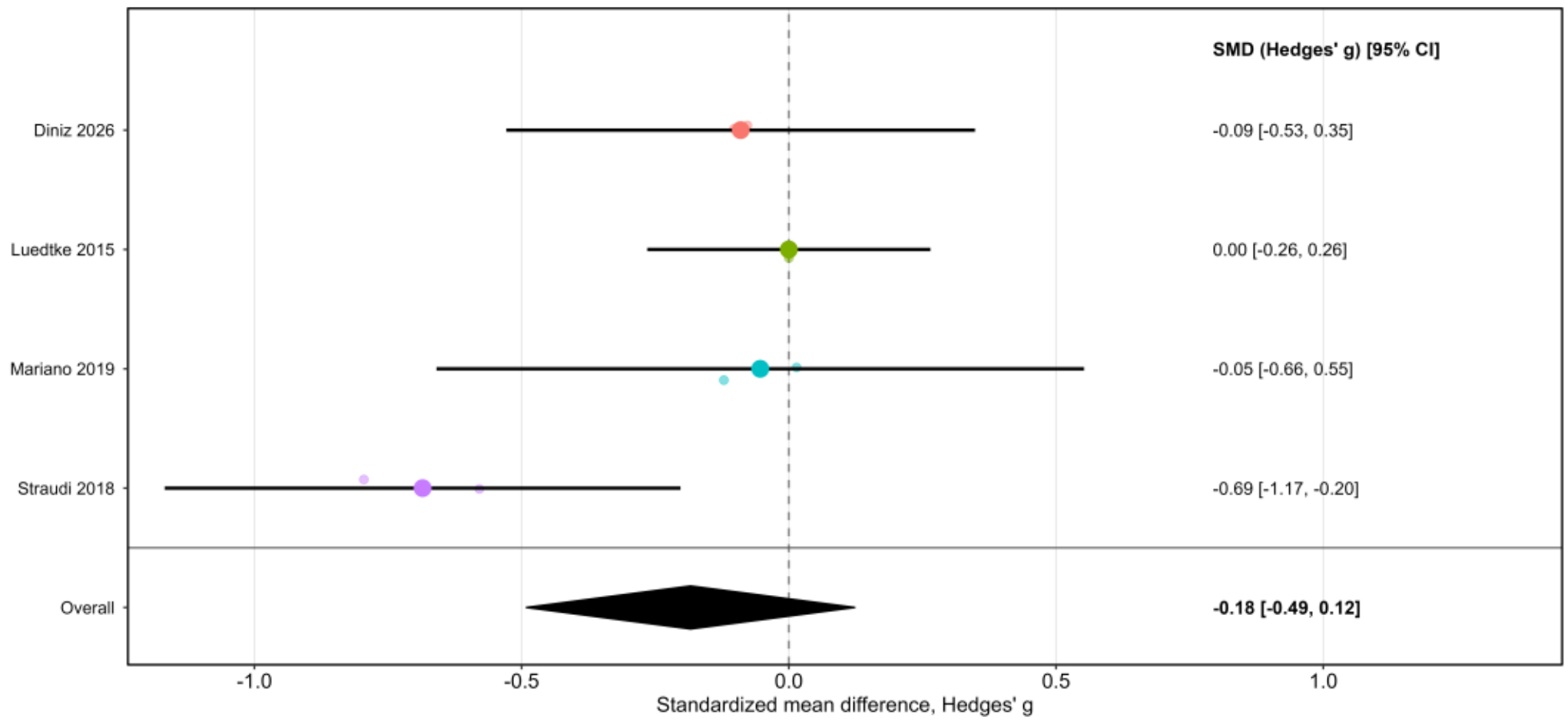

Figure S4: Sensitivity analyses using alternative correlation coefficients

Figure S4-1 Sensitivity analysis of the meta-analysis results assuming r = 0.3

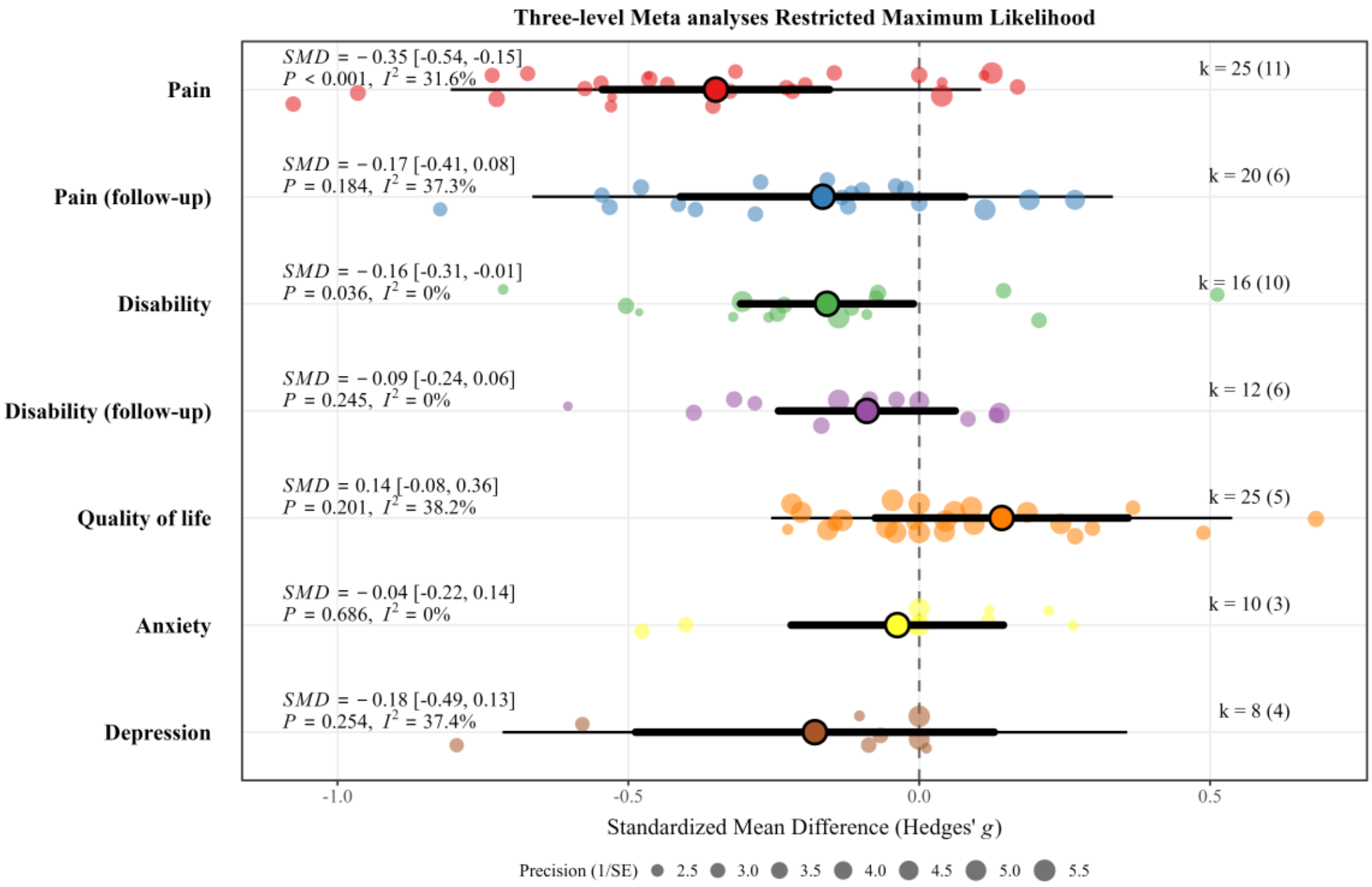

Figure S4-2 Sensitivity analysis of the meta-analysis results assuming r = 0.7

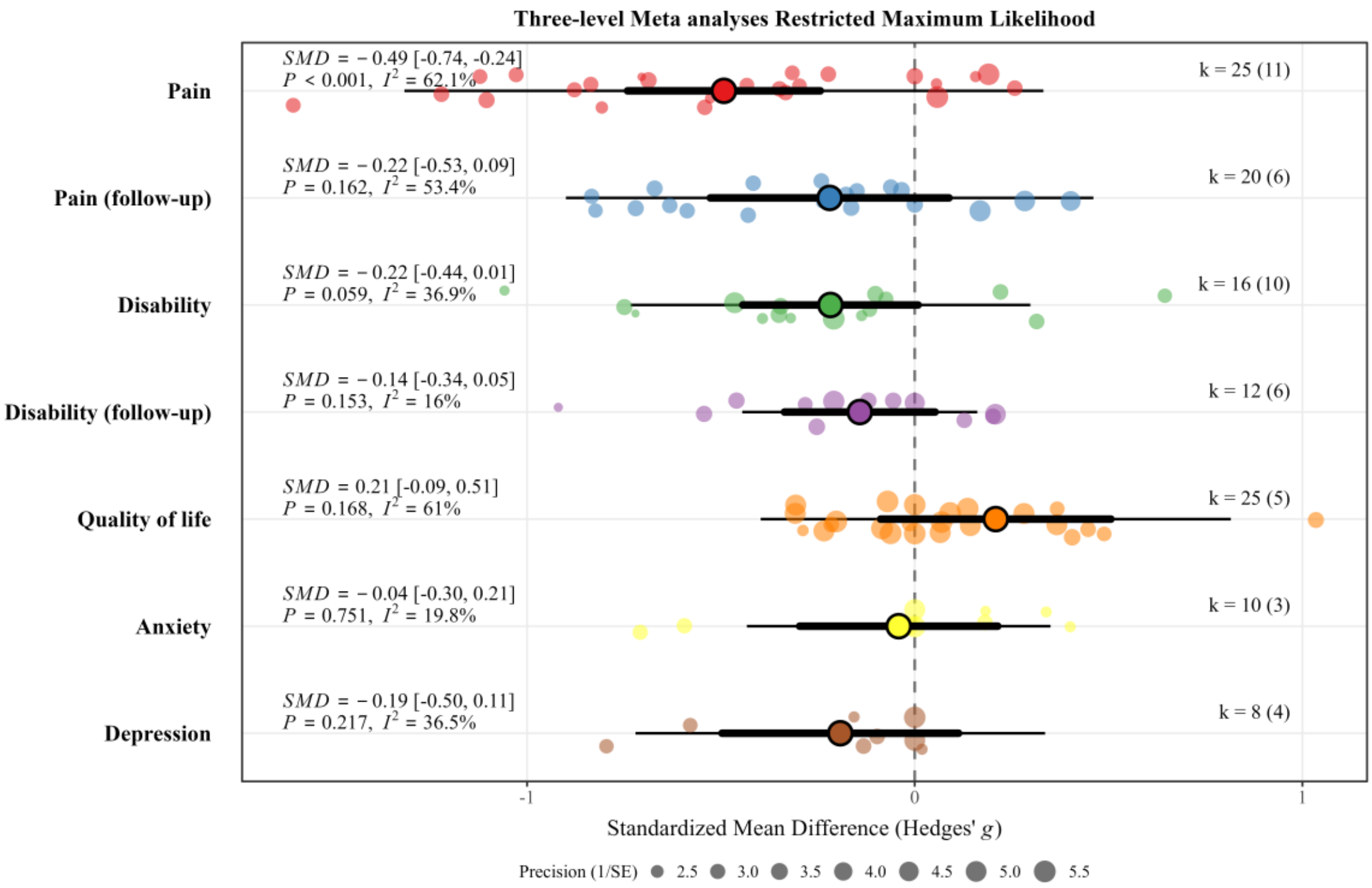

Figure S5: Sensitivity analyses

Figure S5-1 Sensitivity analysis for pain

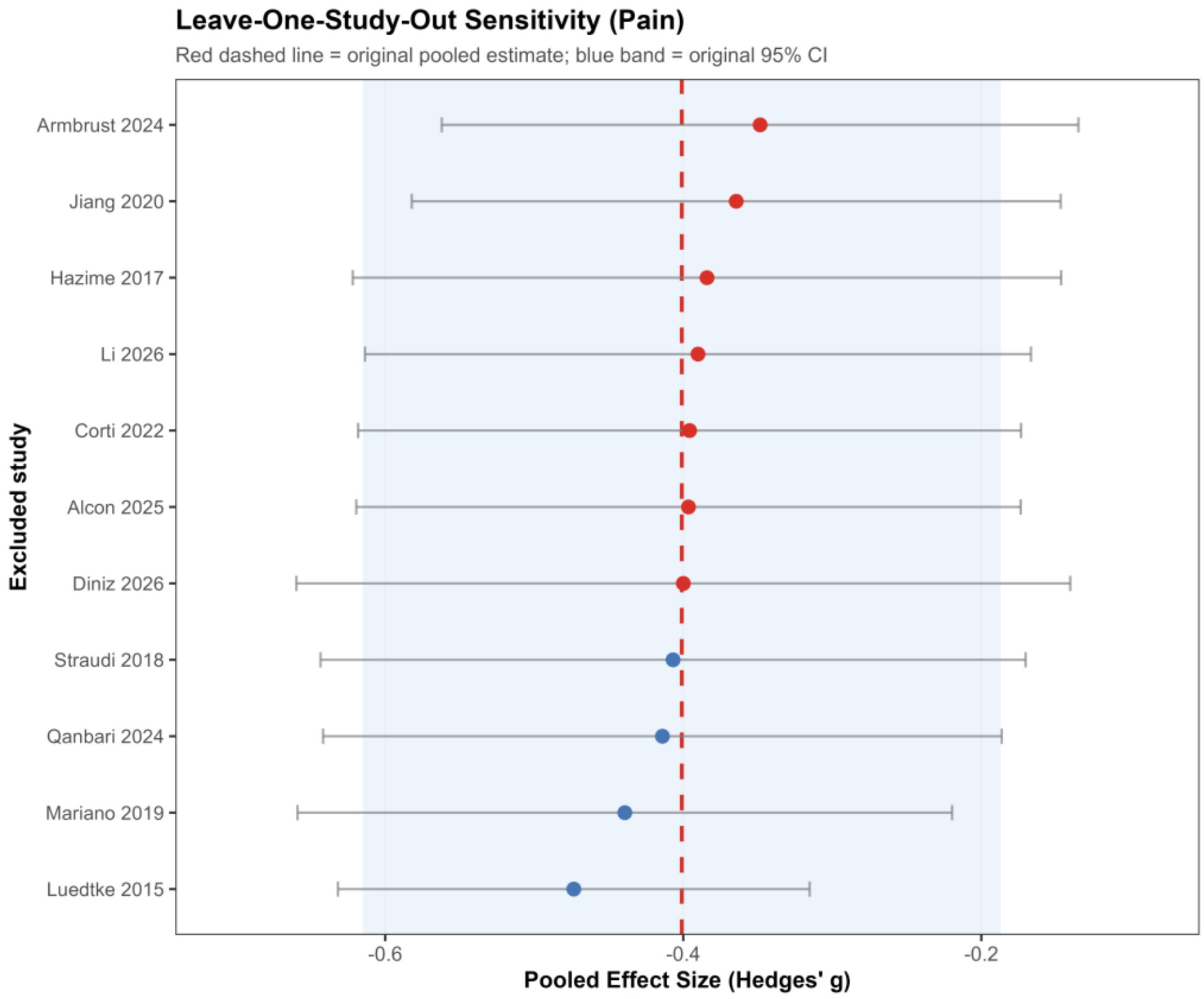

Figure S5-2 Sensitivity analysis for follow-up pain

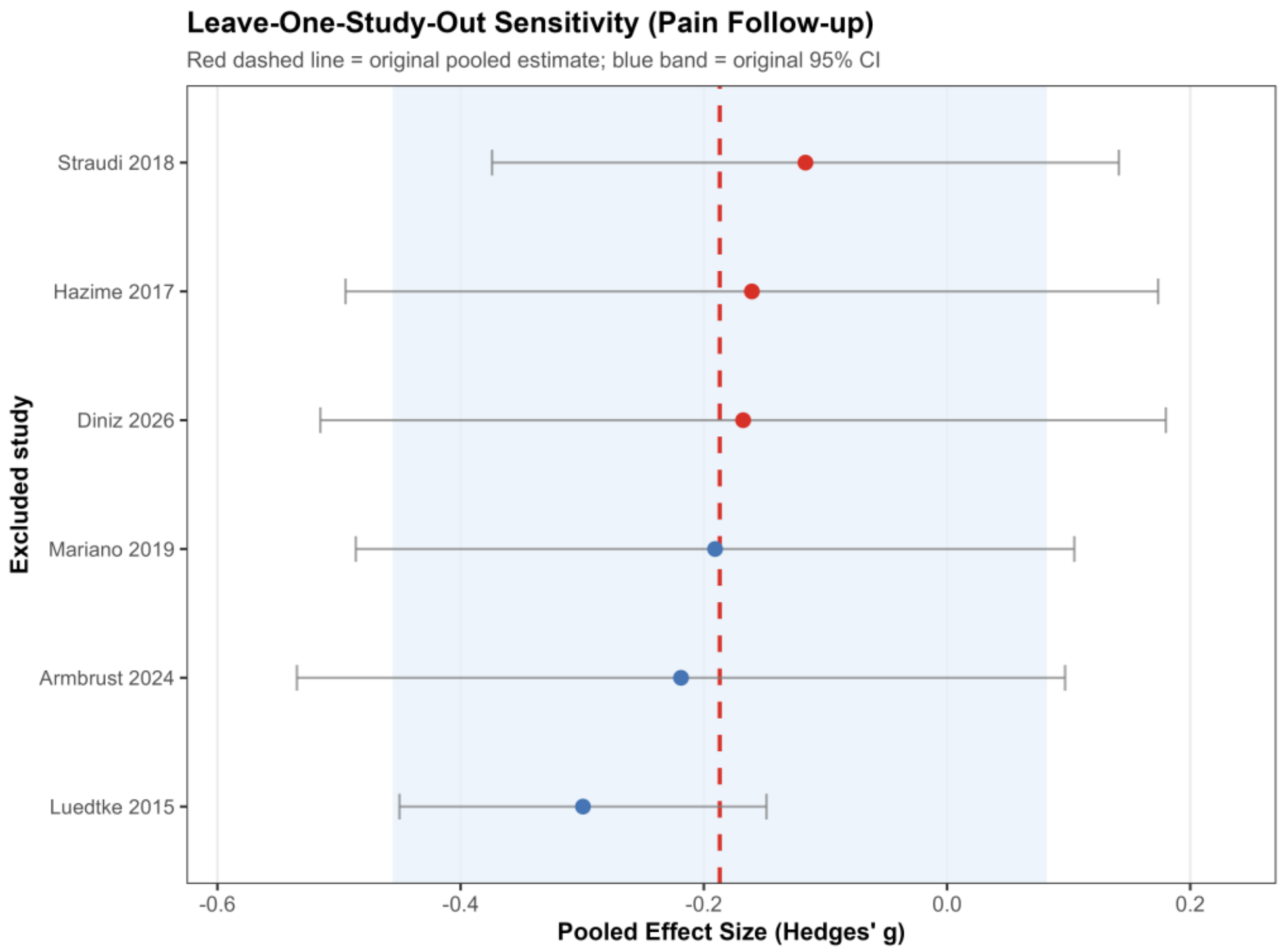

Figure S5-3 Sensitivity analysis for disability

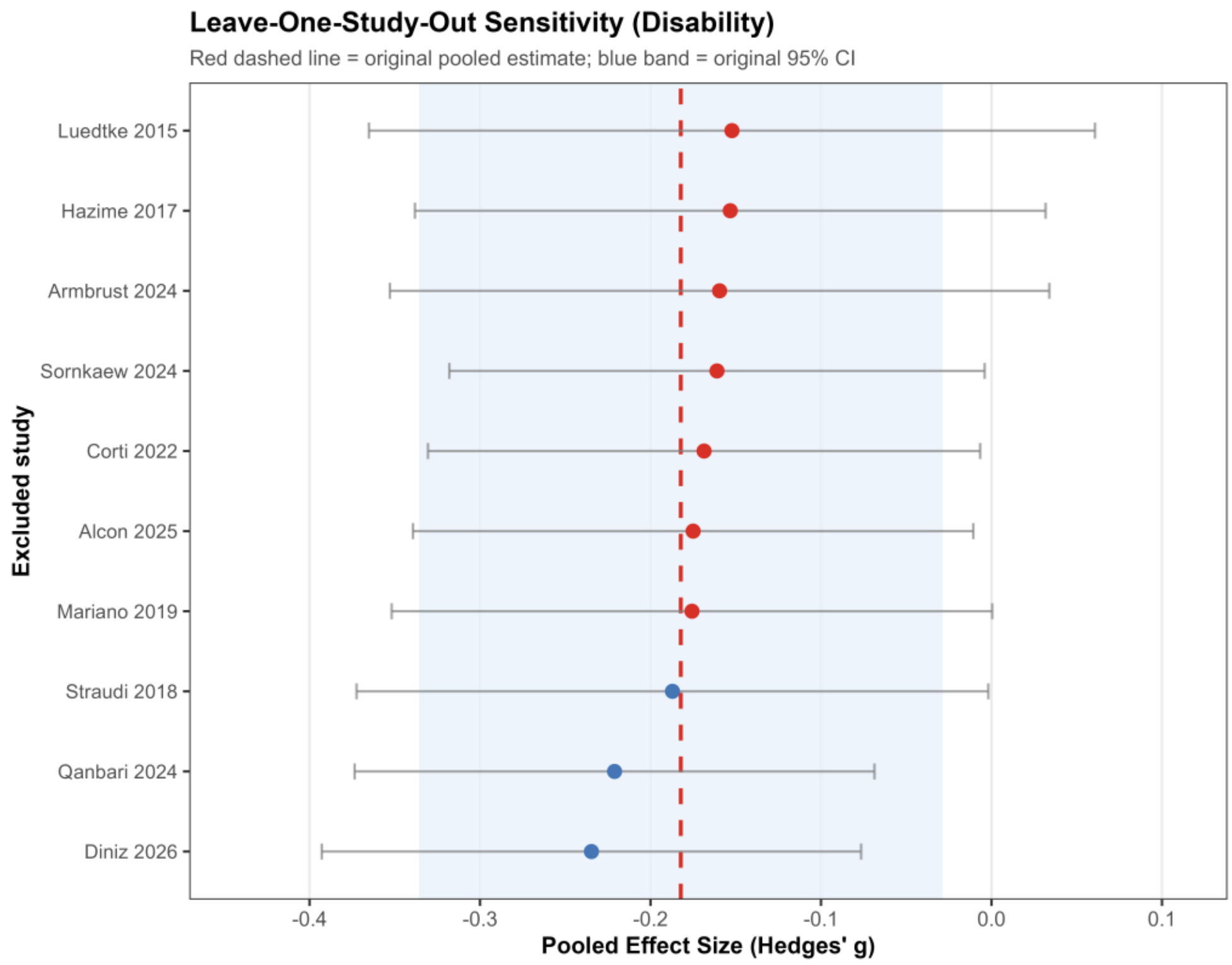

Figure S5-4 Sensitivity analysis for follow-up disability

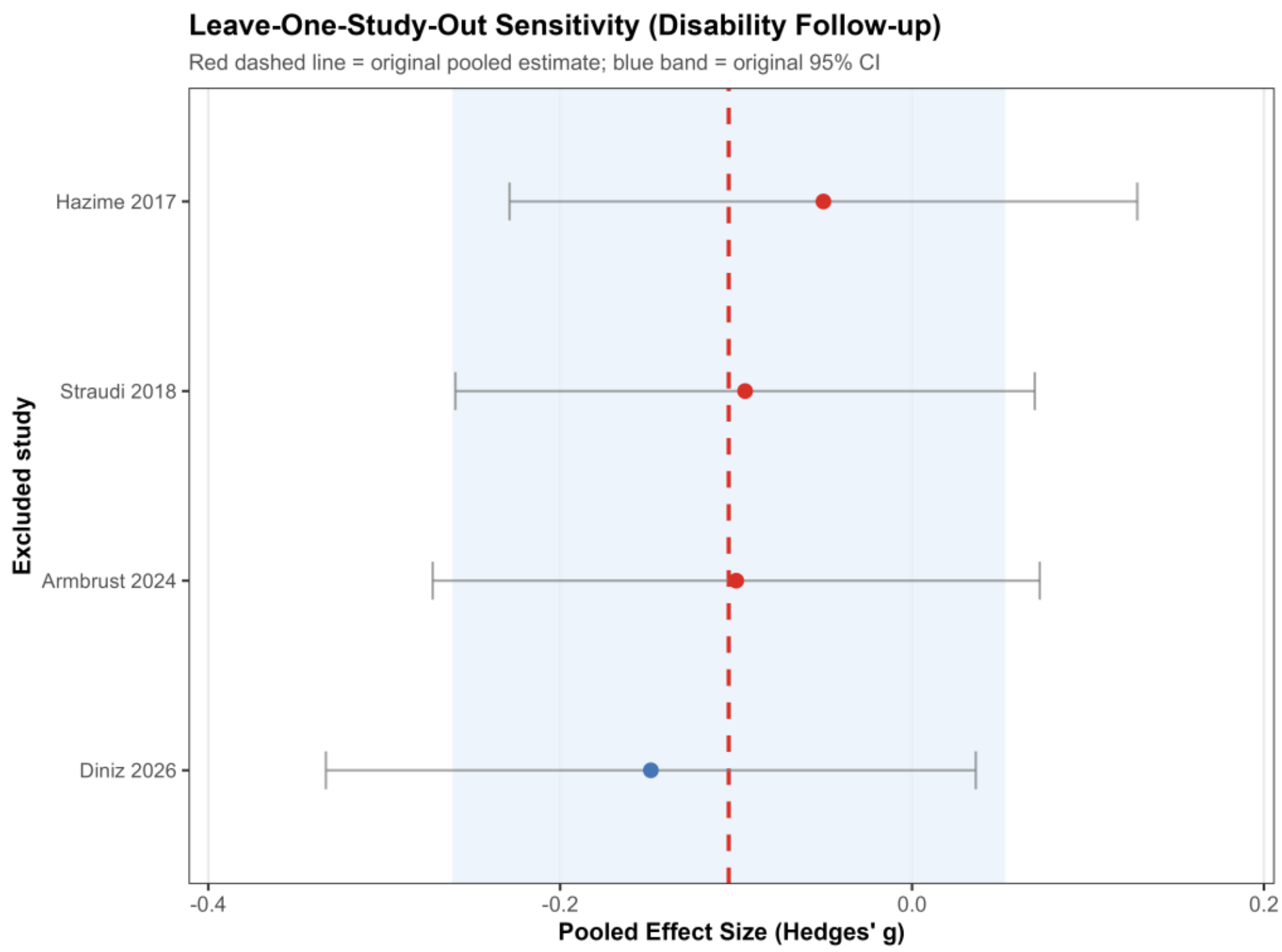

Figure S5-5 Sensitivity analysis for quality of life

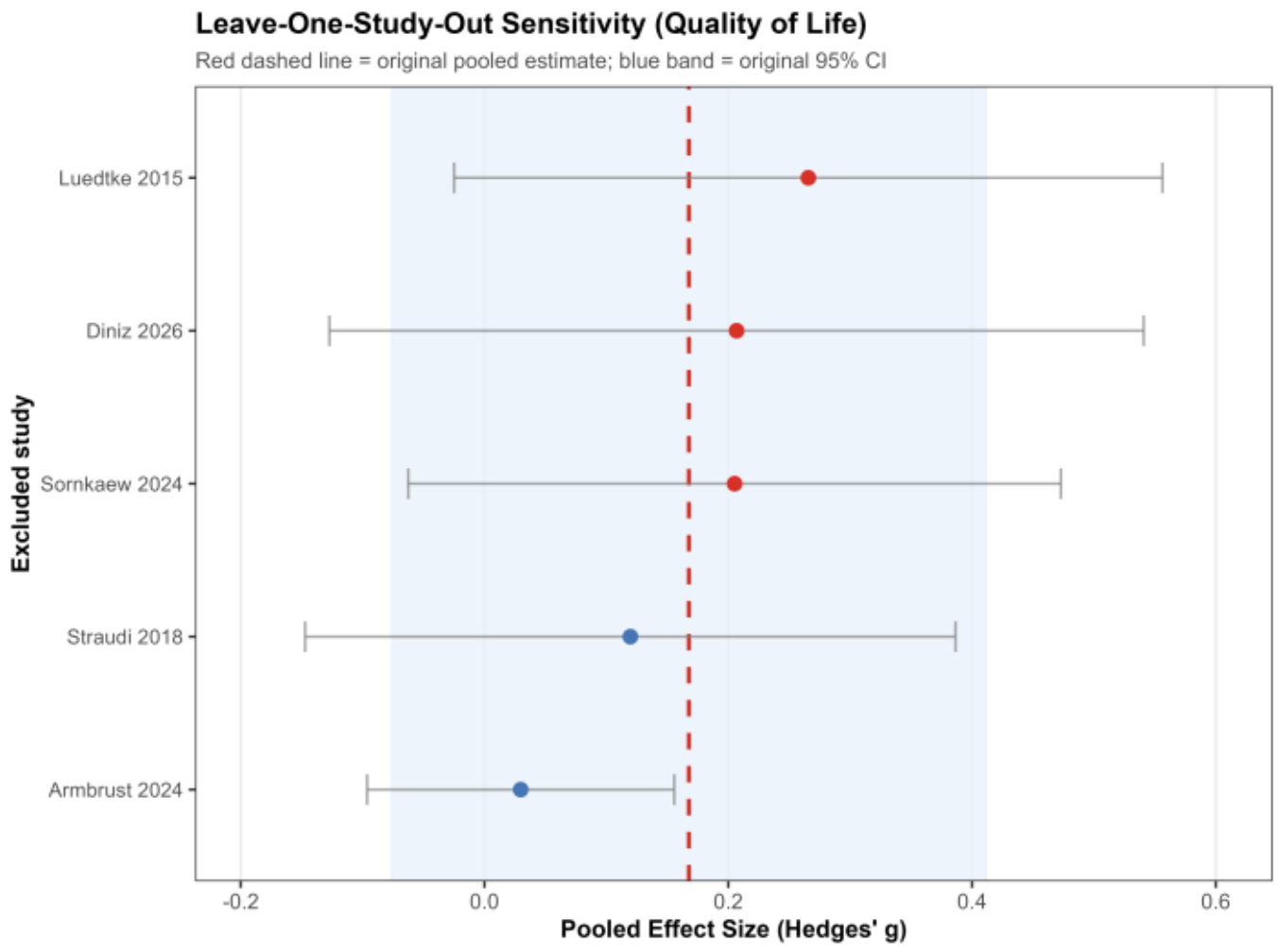

Figure S5-6 Sensitivity analysis for anxiety

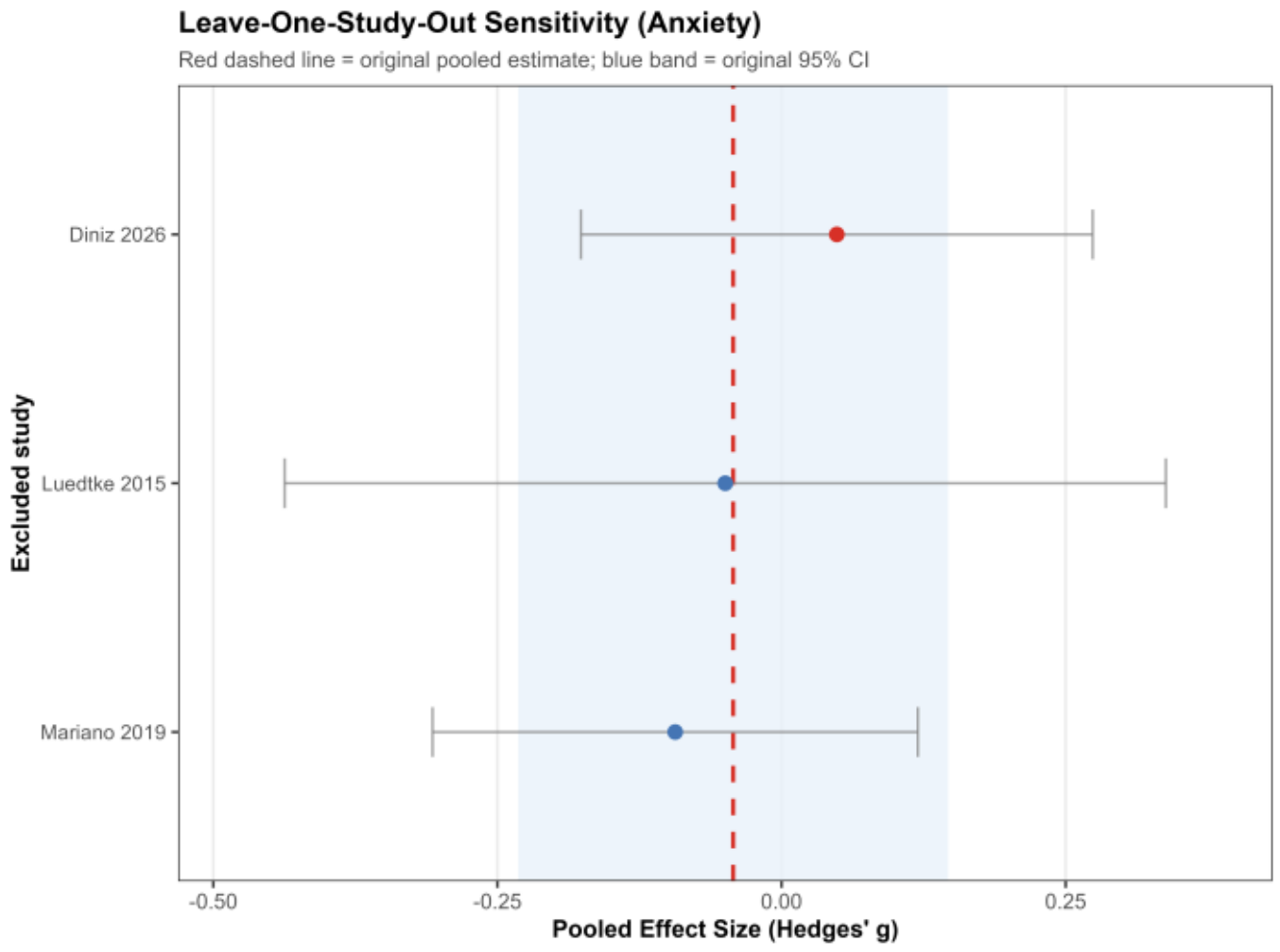

Figure S5-7 Sensitivity analysis for depression

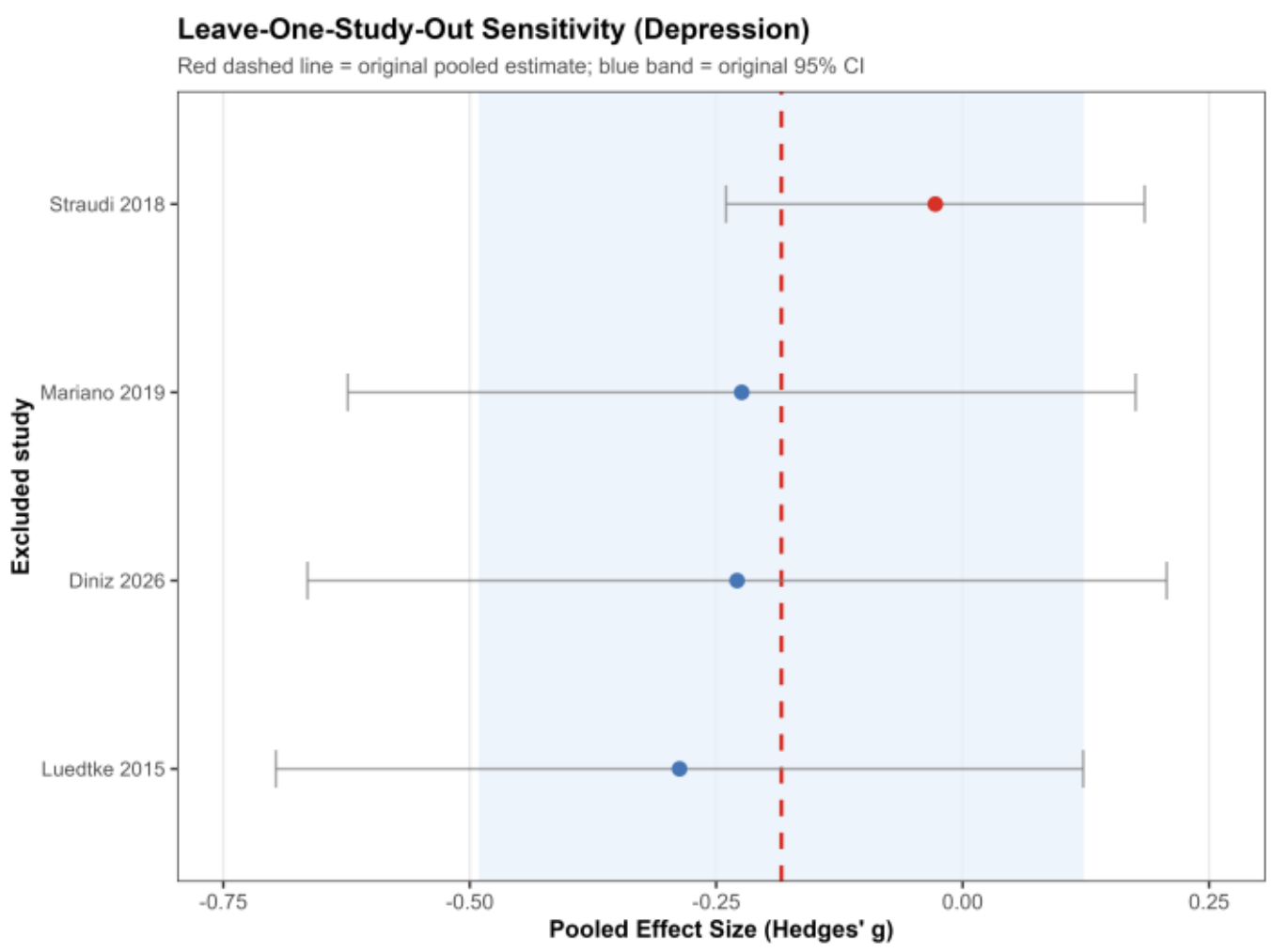

Figure S6: Publication bias

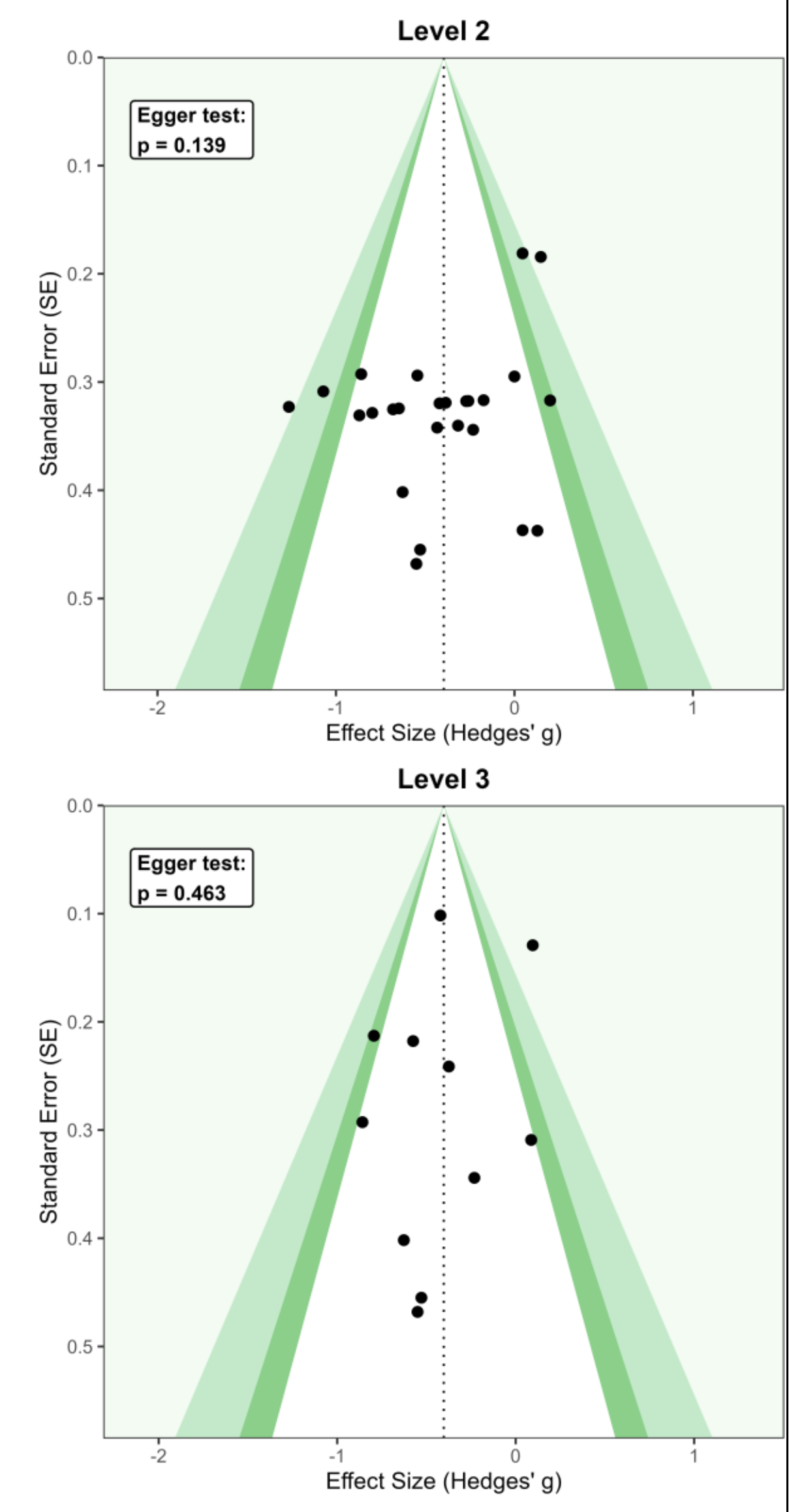

Figure S6-1 Publication bias for pain

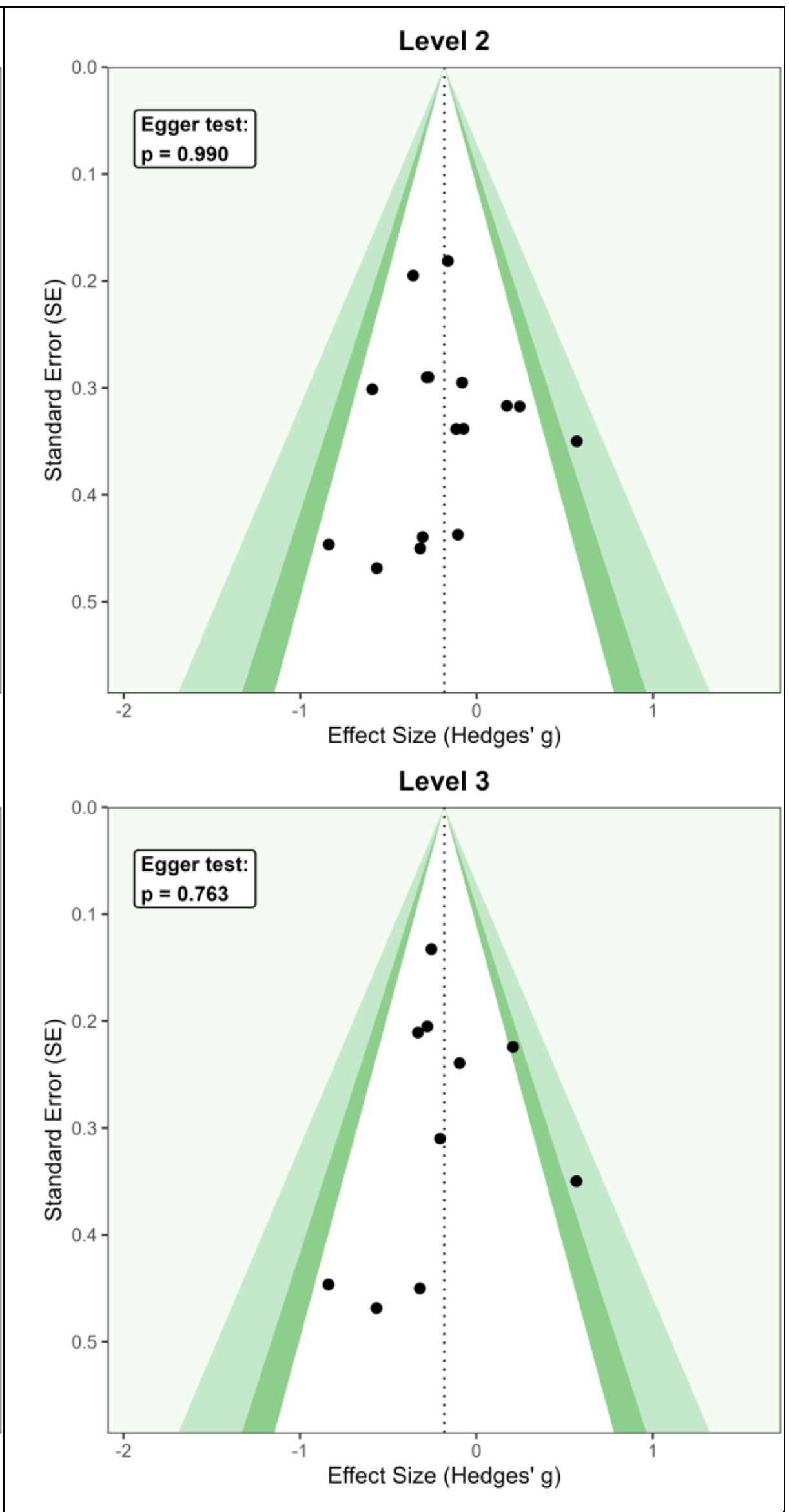

Figure S6-2 Publication bias for disability

References

[1] Alcon C, Zoch C, Luetkenhaus R, Lyman E, Brizzolara K, Goh HT, Wang-Price S. Combined transcranial direct current stimulation and pain neuroscience education for chronic low back pain: a randomized controlled trial. Pain Med 2025;26(2):90-97. doi: 10.1093/pm/pnae101.

[2] Armbrust D, Arêas GPT, Fonseca CL, Arêas F, Duarte NAC, Santana SAA, Dumont AJL, Neto HP, Oliveira CS. Effects of osteopathic manipulative treatment associated with transcranial direct current stimulation in individuals with chronic low back pain: A double-blind, randomised placebo-controlled trial. Clin Rehabil 2024;38(12):1609-1621. doi: 10.1177/02692155241274718.

[3] Corti EJ, Nguyen AT, Marinovic W, Gasson N, Loftus AM. Anodal-TDCS over Left-DLPFC Modulates Motor Cortex Excitability in Chronic Lower Back Pain. Brain Sci 2022;12(12). doi: 10.3390/brainsci12121654.

[4] Diniz LSM, Mousinho RRdS, Cavalcante LL, Andrade SMMdS, Santos HHd, Andrade PRd. Effects of TDCS and TENS on chronic low back pain: A randomized controlled clinical trial. J Bodyw Mov Ther 2026;46:267-273. doi: 10.1016/j.jbmt.2025.10.044.

[5] Hazime FA, Baptista AF, de Freitas DG, Monteiro RL, Maretto RL, Hasue RH, João SMA. Treating low back pain with combined cerebral and peripheral electrical stimulation: A randomized, double-blind, factorial clinical trial. Eur J Pain 2017;21(7):1132-1143. doi: 10.1002/ejp.1037.

[6] Jiang N, Wei J, Li G, Wei B, Zhu FF, Hu Y. Effect of dry-electrode-based transcranial direct current stimulation on chronic low back pain and low back muscle activities: A double-blind sham-controlled study. Restor Neurol Neurosci 2020;38(1):41-54. doi: 10.3233/rnn-190922.

[7] Li Y, Xie H, Peng J, Xu Z, Fu R, Cheng X, Yang J, Yu Q, Xie H, Wang X, Wang C. The immediate effect of anodal tDCS over the motor cortex on postural control and cortical activation in patients with chronic low back pain: A preliminary study. Behav Brain Res 2026;496:115827. doi: 10.1016/j.bbr.2025.115827.

[8] Loan Pham Thi M, Donpunha W, Nakmareong S, Auvichayapat P, Sawanyawisuth K, Janyacharoen T. Effects of transcranial direct-current stimulation combined with physical therapy in chronic low back pain treatments: A randomized controlled pilot study. Chula Med J 2022;66(2):189-196.

[9] Luedtke K, Rushton A, Wright C, Jürgens T, Polzer A, Mueller G, May A. Effectiveness of transcranial direct current stimulation preceding cognitive behavioural management for chronic low back pain: sham controlled double blinded randomised controlled trial. Bmj 2015;350:h1640. doi: 10.1136/bmj.h1640.

[10] Mariano TY, Burgess FW, Bowker M, Kirschner J, Van't Wout-Frank M, Jones RN, Halladay CW, Stein M, Greenberg BD. Transcranial Direct Current Stimulation for Affective Symptoms and Functioning in Chronic Low Back Pain: A Pilot Double-Blinded, Randomized, Placebo-Controlled Trial. Pain Med 2019;20(6):1166-1177. doi: 10.1093/pm/pny188.

[11] Qanbari S, Khanmohammadi R, Olyaei G, Hosseini Z, Hejazi HS. Effects of combining sensory-motor exercises with transcranial direct current stimulation on cortical processing and clinical symptoms in patients with lumbosacral radiculopathy: An exploratory randomized controlled trial. PLoS One 2024;19(12):e0314361. doi: 10.1371/journal.pone.0314361.

[12] Shiasy Y, Shakiba S, Taremian F, Akhavan Hejazi SM, Abasi A. The Effectiveness of Attention Bias Modification with and without Trans Cranial Direct Current Stimulation in Chronic Low Back Pain. Iran J Psychiatry 2020;15(2):112-125.

[13] Sornkaew K, Thu KW, Silfies SP, Klomjai W, Wattananon P. Effects of combined anodal transcranial direct current stimulation and motor control exercise on cortical topography and muscle activation in individuals with chronic low back pain: A randomized controlled study. Physiother Res Int 2024;29(3):e2111. doi: 10.1002/pri.2111.

[14] Straudi S, Buja S, Baroni A, Pavarelli C, Pranovi G, Fregni F, Basaglia N. The effects of transcranial direct current stimulation (tDCS) combined with group exercise treatment in subjects with chronic low back pain: a pilot randomized control trial. Clin Rehabil 2018;32(10):1348-1356. doi: 10.1177/0269215518777881.
